# Clinical and genome-wide association analysis of chemoradiation-induced hearing loss in nasopharyngeal carcinoma

**DOI:** 10.1007/s00439-023-02554-0

**Published:** 2023-04-16

**Authors:** Yong-Qiao He, Lu-Ting Luo, Tong-Min Wang, Wen-Qiong Xue, Da-Wei Yang, Dan-Hua Li, Hua Diao, Ruo-Wen Xiao, Chang-Mi Deng, Wen-Li Zhang, Ying Liao, Yan-Xia Wu, Qiao-Ling Wang, Ting Zhou, Xi-Zhao Li, Xiao-Hui Zheng, Pei-Fen Zhang, Shao-Dan Zhang, Ye-Zhu Hu, Ying Sun, Wei-Hua Jia

**Affiliations:** 1 State Key Laboratory of Oncology in South China, Collaborative Innovation Center for Cancer Medicine, Guangdong Key Laboratory of Nasopharyngeal Carcinoma Diagnosis and Therapy, Sun Yat-sen University Cancer Center, Guangzhou, Guangdong 510060 People’s Republic of China; 2 School of Public Health, Sun Yat-sen University, Guangzhou, People’s Republic of China; 3 Biobank of Sun Yat‑sen University Cancer Center, Guangzhou, People’s Republic of China; 4 Department of Radiation Oncology, State Key Laboratory of Oncology in South China, Collaborative Innovation Center for Cancer Medicine, Guangdong Key Laboratory of Nasopharyngeal Carcinoma Diagnosis and Therapy, Sun Yat-sen University Cancer Center, Guangzhou, People’s Republic of China

## Abstract

Chemoradiation-induced hearing loss (CRIHL) is one of the most devasting side effects for nasopharyngeal carcinoma (NPC) patients, which seriously affects survivors’ long-term quality of life. However, few studies have comprehensively characterized the risk factors for CRIHL. In this study, we found that age at diagnosis, tumor stage, and concurrent cisplatin dose were positively associated with chemoradiation-induced hearing loss. We performed a genome-wide association study (GWAS) in 777 NPC patients and identified rs1050851 (within the exon 2 of *NFKBIA*), a variant with a high deleteriousness score, to be significantly associated with hearing loss risk (HR = 5.46, 95% CI 2.93–10.18, *P* = 9.51 × 10^–08^). The risk genotype of rs1050851 was associated with higher *NFKBIA* expression, which was correlated with lower cellular tolerance to cisplatin. According to permutation-based enrichment analysis, the variants mapping to 149 hereditary deafness genes were significantly enriched among GWAS top signals, which indicated the genetic similarity between hereditary deafness and CRIHL. Pathway analysis suggested that synaptic signaling was involved in the development of CRIHL. Additionally, the risk score integrating genetic and clinical factors can predict the risk of hearing loss with a relatively good performance in the test set. Collectively, this study shed new light on the etiology of chemoradiation-induced hearing loss, which facilitates high-risk individuals’ identification for personalized prevention and treatment.

**Supplementary Information:**

The online version contains supplementary material available at 10.1007/s00439-023-02554-0.

## Introduction

Platinum compounds are the most widely used chemotherapy agents for cancer worldwide, but are also known for their serious side effects of ototoxicity. In addition, radiotherapy to head and neck tumors, such as nasopharyngeal carcinoma (NPC), often inevitably damages the adjacent organs of auditory, which may further increase the risk of hearing loss (HL). Previous studies reported that both platinum and radiation could contribute to DNA damage to auditory cells (Mujica-Mota et al. 2014), leading to apoptosis and death of hair cells, stria vascularis endothelial cells, and spiral ganglion neurons (Landier 2016; Shi et al. 2021). Platinum-based concurrent chemoradiotherapy has been recommended as the standard treatment modality for advanced NPC (Pfister et al. 2020), a malignancy particularly prevalent in the east and southeast Asia, especially in Southern China (Chen et al. 2019). Therefore, the side effect of chemoradiation-induced hearing loss (CRIHL) is particularly devasting for NPC patients (Low et al. 2006), with 21–34% developing hearing impairment (Wu et al. 2014; Tang et al. 2018; Zhang et al. 2019b). However, since the lack of effective treatment, affected patients suffered from a progressive and persistent process of hearing loss, often leading to dramatic declines in their quality of life.

Several clinical factors, including age (Kwong et al. 1996; Wang et al. 2015), cochlea radiation dose, and cisplatin dose (Chen et al. 2006; Chan et al. 2009; Wang et al. 2015), have been reported to be associated with chemotherapy or radiotherapy-related ototoxicity. Nevertheless, these clinical parameters could not fully account for inter-patient variability, suggesting there remain other potential factors playing roles in individual predisposition to treatment-induced hearing loss. Genome-wide association studies (GWASs) have reported that genetic variants were associated with cisplatin or radiation-induced ototoxicity in pediatric cancer survivors (mainly medulloblastoma, osteosarcoma, and acute lymphoblastic leukemia) or adult testicular patients (Xu et al. 2015; Wheeler et al. 2017; Meijer et al. 2021; Trendowski et al. 2021), indicating the underlying role of genetic variants in cisplatin or radiation-induced hearing loss. However, previous GWASs were mainly conducted in European populations of pediatric patients and those with certain cancer types, only focusing on the risk of hearing loss induced by cisplatin or radiotherapy alone. Researches to investigate the genetic backgrounds for hearing loss resulting from chemotherapy in combination with radiotherapy in other ancestries were limited. Especially for NPC, which is treated with a combined modality of chemoradiotherapy and has an extremely unbalanced ethnical distribution, the etiology of CRIHL has never been fully studied yet.

To comprehensively characterize the risk factors for CRIHL, we evaluated the influence of clinical factors and identified the genetic factors in NPC patients who underwent combined chemoradiotherapy. We think the current study would be helpful to identify high-risk individuals for personalized prevention and treatment for CRIHL.

## Materials and methods

### Patient recruitment and follow-up

A total of 1278 histologically confirmed NPC patients, without distant metastatic at diagnosis, were recruited from Sun Yat-sen University Cancer Center (SYSUCC), between 2009 and 2015. Of these 1278 patients, 21 patients younger than 18 years old at diagnosis, 265 patients with self-reported hearing loss before cancer treatment, and 215 patients without concurrent chemoradiotherapy were excluded. Then, a total of 777 eligible patients were included in the present study. All 777 patients in this study underwent concurrent chemoradiotherapy, which has been recommended as the standard treatment protocol for advanced NPC based on the National Comprehensive Cancer Network guidelines (Pfister et al. 2020). And all patients underwent a standardized intensity-modulated radiotherapy (IMRT) with a prescribed total dose of 66–72 Gy in 28–33 fractions (Wen et al. 2021). Most of patients (704/777) received platinum-based concurrent chemoradiotherapy with/without induction chemotherapy and/or adjuvant chemotherapy. Induction chemotherapy mainly consisted of cisplatin (80–100 mg/m^2^) plus docetaxel and/or fluorouracil every 3 weeks for 2–3 cycles (Yao et al. 2017). Concurrent chemotherapy was mainly cisplatin administered weekly (30–40 mg/m^2^, for 4–6 cycles) or triweekly (80–100 mg/m^2^, for 2–3 cycles) during radiation therapy (Yao et al. 2017).

After completion of treatment, patients were followed up every 3 months during the first 3 years and then every 6 months. In addition to completing clinical examination and laboratory testing, patient-reported adverse events were recorded during outpatient clinic follow-up visits. Patients reporting the event of hearing loss in outpatient clinic follow-up records documented by physicians were defined as cases with hearing loss, whereas those without reported hearing loss were classified as the non-hearing loss group. The details for the collection of other clinical data are described in the Supplementary file 1. The time to hearing loss event was defined as the interval between the initiation of radiotherapy or chemotherapy and the time when the event of hearing loss was first documented. The follow-up time was measured from the start of cancer treatment to the date of the last outpatient clinic follow-up. The Institutional Review Board of Sun Yat-sen University Cancer Center approved this study. A brief overview of study design is presented in Fig. S1.

### Genotyping, imputation and quality control

DNA was isolated from peripheral blood samples using a commercial DNA extraction kit (Qiagen). Genotyping was performed on the Illumina Infinium Global Screening Array. A standard quality control protocol for genotypes was implemented in PLINK v1.9. The detailed information about genotyping and quality control was described in our previous study (He et al. 2022). Individuals with call rate < 95%, gender discrepancy, extreme heterozygosity rate (inbreeding coefficient F > 6 SDs), or pairwise identify by descent (IBD) > 0.25 were removed from the analysis. Besides, genetic principal component analysis (PCA) was applied to identify population stratification outliers (> 6 SDs on any one of the top 10 PCs using five iterations) based on EIGENSTRAT (Price et al. 2006). SNPs on autosomal chromosomes were included, but those with call rate < 95%, minor allele frequency (MAF) < 0.01 or deviating from Hardy-Weinberg equilibrium (HWE) (*P* < 1.0 × 10^–12^) were removed. Imputation was performed with SHAPEIT2 and IMPUTE2, using 1000 Genome Phase III integrated variant set of the entire population as a reference panel. Further quality control was conducted and SNPs with INFO < 0.8, call rate < 95%, or MAF < 0.01 were excluded. Finally, a total of 777 subjects and 5,248,879 variants were retained for subsequent analysis.

### Genome-wide association study

We performed a genome-wide association analysis in 777 patients to identify potential genetic variants by assessing the associations between 5,248,879 SNPs and risk of hearing loss, adjusting for age at diagnosis, T stage, concurrent cisplatin dose, and the first two genetic principal components. Manhattan plot and quantile–quantile plot were generated for showing the overall significance of GWAS. The genomic inflation factor (λ_GC_) was calculated to evaluate the deviation of the observed versus the expected distribution of *P*-values. Kaplan–Meier curve was drawn to illustrate the relationships between SNP genotypes and CRIHL after adjusting for the same covariates aforementioned. Additionally, since cisplatin is the most ototoxic platinum compound (Romano et al. 2020) and the main agent used in NPC chemotherapy, we conducted further subgroup analysis in 516 patients treated with cisplatin-based chemotherapy to explore the robustness of associations for the SNPs.

### Permutation-based enrichment analysis

We performed permutation tests to explore the genetic similarity between hereditary hearing loss and chemoradiation-induced hearing loss by examining the enrichment of the genetic variants mapping to the reported hereditary hearing loss genes among GWAS top signals. 149 hereditary deafness genes have been reported to be associated with autosomal non-syndromic and/or syndromic hearing loss from the *Hereditary Hearing Loss Homepage* (https://hereditaryhearingloss.org/). Since approximately 90% of linkage disequilibrium (LD) blocks in the Asian population were observed within the 50 kb gene boundary extension (Wang et al. 2007), we selected 50 kb as the gene boundary for the permutation-based enrichment analysis, which is also referred to the previous studies (Hoffmann et al. 2016; Ghisdal et al. 2017; Wheeler et al. 2017). Then, 56,040 genetic variants within 50 kb of these 149 genes were included for further analysis. In each shuffle, the patient phenotypes, including age at diagnosis, T stage, concurrent cisplatin dose, and ototoxicity (as a time-to-event variable), were randomly permuted relative to the patient genotypes and the association analyses were repeated on the permuted resamples. We counted the number of deafness gene SNPs with a range of significance thresholds from *P* < 5.0 × 10^–02^ to *P* < 5.0 × 10^–06^ in association test for each replicate to examine the robustness of enrichment analysis. 1000 permutation shuffles were conducted to construct the null distribution. The distribution of the number of significant genetic variants within 50 kb of deafness genes was displayed in a histogram, compared with the observed number in the current study. The empirical *P*-value was obtained by calculating the probability of detecting the expected number of significant SNPs larger than or equal to the observed number.

### Functional annotations

We conducted functional annotations for variants in moderate to high LD (*r*^2^ ≥ 0.6) with the lead variants by FUMA (https://fuma.ctglab.nl/). The LD between genetic variants was calculated on the basis of 1000 Genomes Phase III East Asian population. We annotated SNPs by performing ANNOVAR gene-based annotation. The details of functional prediction scores, chromatin marks, and expression quantitative trait loci (eQTL) analysis are shown in the Supplementary file 1.

### Gene expression and cellular sensitivity to cisplatin or radiation

To determine whether the expression of susceptible genes exerts effects on chemosensitivity, we examined the relationship between gene expression and cellular sensitivity to cisplatin in the central nervous system (CNS) cancer cell lines (mainly glioma cell lines), as well as other cancer cell lines across 11 histology types (bladder/urinary tract, bone, bowel, breast, kidney, lung, ovary/fallopian tube, pancreas, peripheral nervous system, skin and soft tissue), which was referred to in previous studies (Wheeler et al. 2017; El Charif et al. 2019) since there is no available relevant data on auditory cell lines. Baseline gene expression data in different cell lines was generated by the Cancer Cell Line Encyclopedia (CCLE) project (Barretina et al. 2012). Cisplatin sensitivity, measured as the concentration of cisplatin inhibiting half of cellular growth (IC_50_), was derived from the Genomics of Drug Sensitivity in Cancer (GDSC) database (Yang et al. 2013). We obtained data on gene expression and cisplatin sensitivity from the curated pharmacogenomic datasets by R package “PharmacoGx” (Smirnov et al. 2016).

Similarly, we examined the correlations of gene expression with radiosensitivity in the cell line types mentioned above (Trendowski et al. 2021). Radiosensitivity was measured by the surviving fraction after 2 Gy radiation (SF2) from the 9-days viability assay in tumor cell lines (Yard et al. 2016). We obtained data on gene expression and radiosensitivity from the curated radiogenomic datasets by R package “RadioGx” (Manem et al. 2019). Associations of gene expression with cellular sensitivity to cisplatin or radiation were estimated by Spearman’s rank correlation test in R 4.1.1.

### Pathway analysis

We conducted pathway enrichment analysis to explore the biological processes involved in the development of CRIHL by R package “clusterProfiler” (Yu et al. 2012). Genes, which had at least one SNP with *P* < 0.001 in the GWAS analysis within their 20 kb upstream/downstream regions, were kept, as the previous study described (Wang et al. 2019). We included 799 candidate genes for pathway enrichment analysis based on Gene Ontology (GO) database. Gene sets with a size less than 10 or more than 500 were excluded from the analysis. Benjamini and Hochberg approach was used to correct for multiple testing.

### Construction of a risk score model

By combining genetic and clinical factors, we constructed a risk score model to estimate the 5-years risk of CRIHL via Cox regression model. Eight SNPs, including seven lead SNPs with *P* < 1.0 × 10^–06^ in the GWAS (rs1050851, rs117098517, rs1485149, rs17010289, rs79938362, rs73335760 and rs201061882) and one lead SNP with *P* < 1.0 × 10^–05^ within the hereditary deafness gene regions (rs2275994), were included in the risk score. The clinical factors of age at diagnosis, tumor stage, and concurrent cisplatin dose were also included in the risk score. All 777 subjects in this study were randomly dichotomized into a training set (*n* = 389) and a test set (*n* = 388). The risk score model was developed based on the samples in the training set and then validated in the test set. We generated the risk score by summing the values weighted by the coefficients of the genetic and clinical variables. The risk score for each patient in this study was calculated, and the median risk score in the training set was regarded as a cutoff value to divide subjects into low or high-risk score groups.

### Statistical analysis

Univariate Cox regression analysis was carried out to test the association of clinical factors with CRIHL. Variables with a two-tailed *P*-value of less than 0.05 in univariate analysis were adjusted in multivariate Cox regression analysis. The statistically significant (*P* < 0.05) clinical factors determined by multivariate analysis were included as covariates in genome-wide association analysis. The optimal cutoff points of continuous variables were defined by receiver operating characteristic (ROC) curve analyses, and categorical variables were classified based on clinical relevance. The GWAS analysis was conducted by Cox proportional hazard model under an additive assumption using the R package “gwasurvivr” (Rizvi et al. 2019). Stratified analysis was conducted to investigate the stability property of association results of the lead variants. All subjects were dichotomized based on age at diagnosis, sex, T stage, pre-treatment EBV DNA levels, treatment modality, primary tumor dose and concurrent cisplatin dose, respectively. In each subgroup, hazard ratio (HR), 95% confidence interval (CI) and *P*-value were estimated via Cox regression model with adjustment for age at diagnosis, T stage and concurrent cisplatin dose (except for itself in the subgroups defined by these variables). All statistical tests were two-sided and performed in R 4.1.1.

## Results

### Patient characteristics

777 eligible NPC patients were recruited and followed up in this study. With a median follow-up time of 70.7 months (IQR 52.7–85.5), 132 (17.0%) patients developed chemoradiation-induced hearing loss. The median time interval from the initiation of cancer treatment to the event of hearing loss occurrence was 18.4 months. The clinical characteristics were shown in Table 1. By univariate Cox regression analyses, age at diagnosis, T stage, induction cisplatin dose, and concurrent cisplatin dose were significantly correlated with CRIHL. By multivariate analyses to identify independent clinical risk factors, we found that elder age (for ≥ 50 years vs. < 50 years at diagnosis: adjusted HR = 1.72, 95% CI 1.19–2.49, *P* = 0.004), advanced tumor stage (for T3–T4 vs. T1–T2: adjusted HR = 1.66, 95% CI 1.12–2.46, *P* = 0.012), and higher concurrent cisplatin dose (for ≥ 155 mg/m^2^ vs. < 155 mg/m^2^: adjusted HR = 1.46, 95% CI 1.01–2.12, *P* = 0.046) were significantly associated with higher risk of hearing loss. However, the other factors of sex, smoking, drinking, pre-treatment EBV DNA loads, radiation dose for the primary tumor, induction cisplatin dose, hypertension, and diabetes were not significantly associated with hearing loss risk.

**Table 1:** Associations of clinical characteristics with chemoradiation-induced hearing loss in 777 nasopharyngeal carcinoma patients

Characteristic	non-HL (*n* = 645)	HL (*n* = 132)	Univariate analysis			Multivariate analysis	
			HR (95% CI)	*P* value		HR (95% CI)^a^	*P* value^a^
Age at diagnosis (years)^b^							
Median (IQR)	42.0 (36.0–49.0)	44.0 (36.0–51.2)	1.01 (0.99–1.03)	0.405		–	–
< 50	501 (77.7%)	90 (68.2%)	Reference			Reference	
≥ 50	144 (22.3%)	42 (31.8%)	1.65 (1.14–2.38)	**0.008**		1.72 (1.19–2.49)	**0.004**
Sex							
Male	466 (72.2%)	88 (66.7%)	Reference			Reference	
Female	179 (27.8%)	44 (33.3%)	1.31 (0.92–1.89)	0.138		1.29 (0.90–1.85)	0.171
T stage							
T1–T2	254 (39.4%)	34 (25.8%)	Reference			Reference	
T3–T4	391 (60.6%)	98 (74.2%)	1.78 (1.20–2.63)	**0.004**		1.66 (1.12–2.46)	**0.012**
N stage							
N0–N1	453 (70.2%)	92 (69.7%)	Reference			Reference	
N2–N3	192 (29.8%)	40 (30.3%)	1.09 (0.75–1.58)	0.647		1.05 (0.72–1.52)	0.816
Overall stage							
I–II	181 (28.1%)	28 (21.2%)	Reference			Reference	
III–IV	464 (71.9%)	104 (78.8%)	1.45 (0.96–2.21)	0.080		0.59 (0.24–1.42)	0.238
EBV DNA loads (copies/ml)							
Median (IQR)	1910 (0–16,150)	3805 (0–22,825)	1.00 (1.00–1.00)	0.575		1.00 (1.00–1.00)	0.536
< 4000	358 (55.5%)	67 (50.8%)	Reference			Reference	
≥ 4000	261 (40.5%)	63 (47.7%)	1.32 (0.93–1.86)	0.119		1.22 (0.86–1.73)	0.261
NA	26 (4.0%)	2 (1.5%)	0.45 (0.11–1.82)	0.259		0.50 (0.12–2.07)	0.342
Treatment modality							
CCRT alone	364 (56.4%)	75 (56.8%)	Reference			Reference	
CCRT + IC/AC	281 (43.6%)	57 (43.2%)	0.99 (0.70–1.39)	0.944		0.72 (0.46–1.12)	0.146
Primary tumor dose (Gy)^b^							
Median (IQR)	70.1 (69.9–70.2)	70.1 (69.9–70.2)	1.02 (0.85–1.21)	0.864		0.94 (0.78–1.13)	0.487
< 70.2	398 (61.7%)	78 (59.1%)	Reference			Reference	
≥ 70.2	247 (38.3%)	54 (40.9%)	1.09 (0.77–1.54)	0.630		1.06 (0.74–1.51)	0.763
Induction cisplatin dose (mg/m^2^)^b^							
Median (IQR); per 100 mg/m^2^ increase	0 (0–139)	0 (0–147)	1.08 (0.88–1.33)	0.476		–	–
< 162	554 (85.9%)	103 (78.0%)	Reference			Reference	
≥ 162	91 (14.1%)	29 (22.0%)	1.59 (1.05–2.40)	**0.027**		1.40 (0.92–2.15)	0.120
Concurrent cisplatin dose (mg/m^2^)^b^							
Median (IQR); per 100 mg/m^2^ increase	160 (0–201)	162 (0–200)	1.10 (0.93–1.31)	0.245		–	–
< 155	279 (43.3%)	43 (32.6%)	Reference			Reference	
≥ 155	366 (56.7%)	89 (67.4%)	1.50 (1.04–2.16)	**0.028**		1.46 (1.01–2.12)	**0.046**
Smoking							
No	455 (70.5%)	92 (69.7%)	Reference			Reference	
Yes	190 (29.5%)	40 (30.3%)	1.05 (0.73–1.53)	0.788		1.00 (0.69–1.45)	0.984
Drinking							
No	560 (86.8%)	116 (87.9%)	Reference			Reference	
Yes	85 (13.2%)	16 (12.1%)	0.90 (0.53–1.52)	0.688		0.98 (0.58–1.65)	0.927
Hypertension							
No	613 (95.0%)	123 (93.2%)	Reference			Reference	
Yes	32 (5.0%)	9 (6.8%)	1.39 (0.71–2.74)	0.339		1.27 (0.64–2.51)	0.494
Diabetes							
No	633 (98.1%)	129 (97.7%)	Reference			Reference	
Yes	12 (1.9%)	3 (2.3%)	1.26 (0.40–3.95)	0.695		1.18 (0.37–3.74)	0.777
Time since therapy initiation to last follow-up (months)							
Median (IQR)	68.9 (51.8–84.3)	75.9 (58.1–89.7)	1.00 (0.99–1.01)	0.965		1.00 (0.99–1.01)	0.560

Abbreviations *HL* hearing loss, *HR* hazard ratio, *CI* confidence interval, *IQR* interquartile range, *CCRT* concurrent chemoradiotherapy, *IC* induction chemotherapy, *AC* adjuvant chemotherapy, *NA* not available

^a^As for multivariate analysis, *P* values, HRs and 95% CIs were estimated via Cox proportional hazard model with the covariates including age at diagnosis, T stage, induction cisplatin dose and concurrent cisplatin dose. *P* values < 0.05 are highlighted in bold

^b^The optimal cutoff points of continuous variables (such as age at diagnosis, primary tumor dose, induction cisplatin dose, and concurrent cisplatin dose) were defined by receiver operating characteristic (ROC) curve analyses

### GWAS analysis for chemoradiation-induced hearing loss

**Fig. 1:**
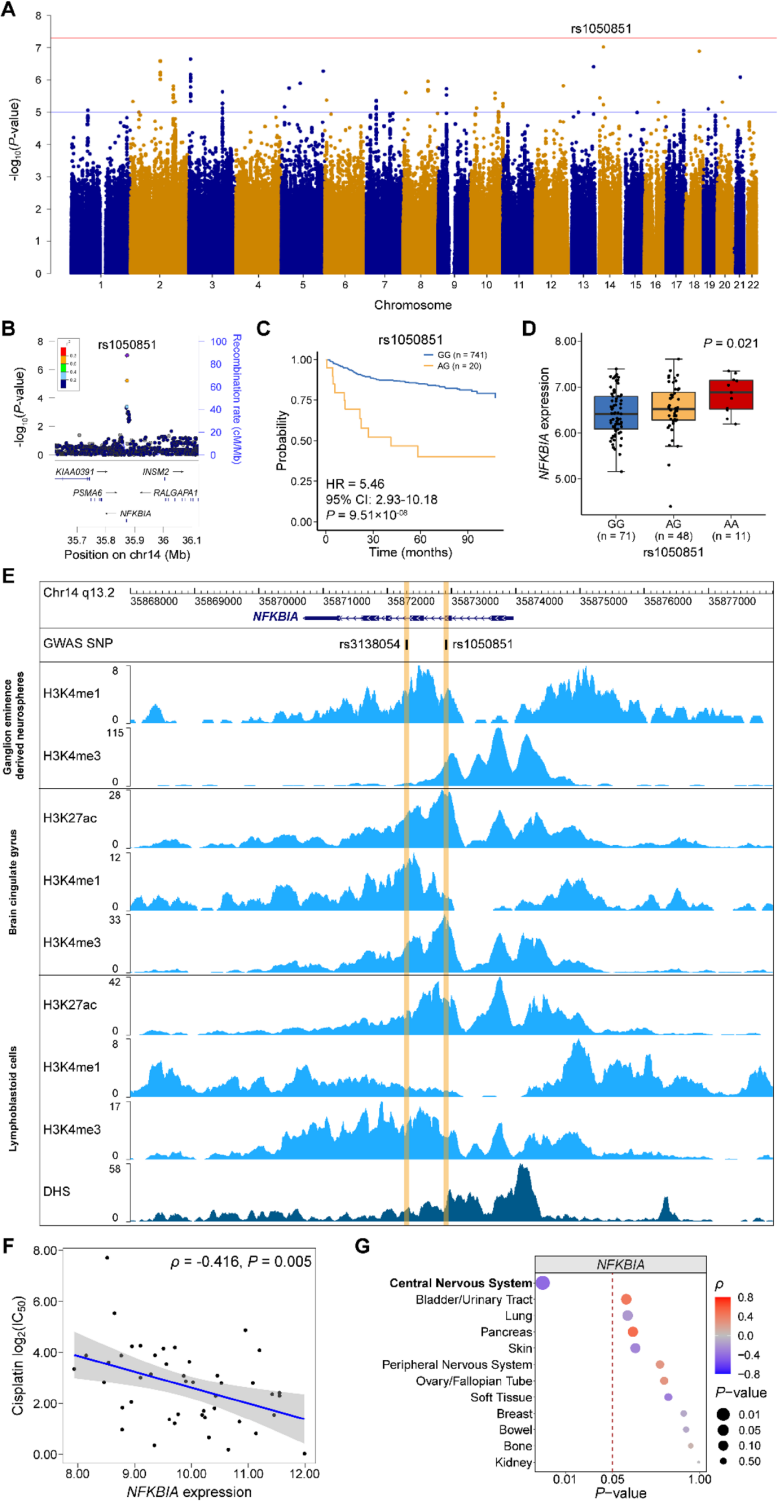
Genome-wide association results of chemoradiation-induced hearing loss and functional annotations on *NFKBIA* locus. **A** The association of SNP genotype and hearing loss in 777 nasopharyngeal carcinoma patients was evaluated via Cox proportional hazard model. The red and blue lines indicate the genome-wide significance threshold (*P* < 5.0 × 10^–08^) and the suggestive threshold (*P* < 1.0 × 10^–05^), respectively. **B** Regional plot of association results for *NFKBIA* locus. The lead variant rs1050851 is marked by a purple diamond. The color of each circle indicates the linkage disequilibrium r^2^ with rs1050851 in 1000 Genome Asian populations. **C** The relationship between rs1050851 genotype and chemoradiation-induced hearing loss (no AA genotype was observed). **D** Boxplot of *NFKBIA* expression in the cerebellar cortex (CRBL) of human brain by rs1050851 genotype. Data was obtained from BRAINEAC database. **E** ChIP-seq data shows enrichments of histone modification marks and DNase I hypersensitive sites in the sites of the lead variant rs1050851 and another variant in strong LD with it (rs3138054, *r*^2^ = 0.78). **F** Scatter plot of cisplatin sensitivity against normalized *NFKBIA* gene expression in central nervous system cancer cell lines. **G** Correlations of cisplatin sensitivity with *NFKBIA* expression in different cancer cell line types. The dashed red line indicates *P*-value of 0.05. *ρ* and *P*-value were calculated using Spearman’s rank correlation

The GWAS was conducted in 777 patients to assess the associations between 5,248,879 SNPs and the risk of hearing loss by the Cox proportional hazard model under an additive assumption. The quantile–quantile plot showed a good match between the distribution of the observed *P*-values and the expected *P*-values by chance (λ_GC_ = 1.01), and an apparent deviation within the tail of the distribution, indicating true associations (Fig. S2). GWAS identified 30 loci surpassing the suggestive threshold (*P* < 1.0 × 10^–05^; Table S1). rs1050851 was the most significant SNP associated with the risk of CRIHL (HR = 5.46, 95% CI 2.93–10.18, *P* = 9.51 × 10^–08^; Fig. 1A and Table S2). Since cisplatin is the most ototoxic platinum compound (Romano et al. 2020) and the main agent used in NPC chemotherapy, we conducted a subgroup analysis in 516 patients who were treated with cisplatin-based chemotherapy and the clinical characteristics were shown in Table S3. We found rs1050851 exceeded the significant threshold of *P* < 5.0 × 10^–08^ (HR = 9.16, 95% CI 4.76–17.64, *P* = 3.32 × 10^–11^; Table S4) in the subgroup patients who received cisplatin-based chemotherapy, indicating the robustness of the association. Patients with the risk allele (A) at rs1050851 were more susceptible to hearing loss in comparison with those carrying the non-risk allele (G) (Fig. 1C). To be specific, the incidence of hearing loss among patients carrying AG genotype at rs1050851 was 55.0%, which was much higher than that of 15.8% among patients carrying GG genotype (no AA genotype was observed).rs1050851 is a synonymous coding variant within the exon 2 of *NFKBIA* gene (Fig. 1B). To measure the deleteriousness of this SNP, we annotated with functional prediction scores and found it has a very high CADD score of 17.10, indicating a deleterious mutation (Table S5). eQTL analysis showed that the risk allele at rs1050851 was correlated with higher expression of *NFKBIA* in human brain tissues (such as the cerebellar cortex; Fig. 1D and Table S6), as well as in blood tissues (Table S7). Similar eQTL associations were also seen between genotype of rs3138054 (*r*^2^ = 0.78 with rs1050851) and *NFKBIA* expression (Fig. S3, Tables S6 and S7). Additionally, both rs1050851 and rs3138054 were located within the regions containing enhancer or promoter histone markers (eg, H3K27ac, H3K4me1, and H3K4me3) in multiple tissues, including ganglion eminence derived neurospheres, brain cingulate gyrus and lymphoblastoid cells, as well as within the DNase I hypersensitive site (DHS) peaks in lymphoblastoid cells (Fig. 1E). These results suggested that rs1050851 and rs3138054 might modulate the expression of *NFKBIA* through affecting the promoter or enhancer activity, or chromatin accessibility, to influence the risk of CRIHL.

By cisplatin and radiation cytotoxicity analysis, we found that higher expression of *NFKBIA* gene was significantly associated with lower cisplatin IC_50_ (that is, lower tolerance to cisplatin) in CNS cancer cell lines (*ρ* = − 0.416, *P* = 0.005; Fig. 1F), while the expression of *NFKBIA* gene was not associated with radiosensitivity in the cell lines (*ρ* = 0.118, *P* = 0.500), indicative of a harmful function of *NFKBIA* against cisplatin-induced injury. *NFKBIA* gene encodes a member of NF-κB inhibitor family, IκBα protein, which is involved in inflammatory responses in the CNS (O'Neill and Kaltschmidt 1997). Interestingly, we found that the significant association of cisplatin sensitivity with *NFKBIA* expression was specific to cancer cell lines from CNS tissues, not from other tissue types (Fig. 1G).

By and large, carriers with the minor allele (A) at rs1050851 would have higher *NFKBIA* expression and lower cisplatin tolerance, being prone to cisplatin-induced injury, which may partially explain the increased hearing loss risk for patients carrying the risk allele at rs1050851 in the GWAS analysis.

### Enrichment analysis of hereditary hearing loss genes

**Fig. 2:**
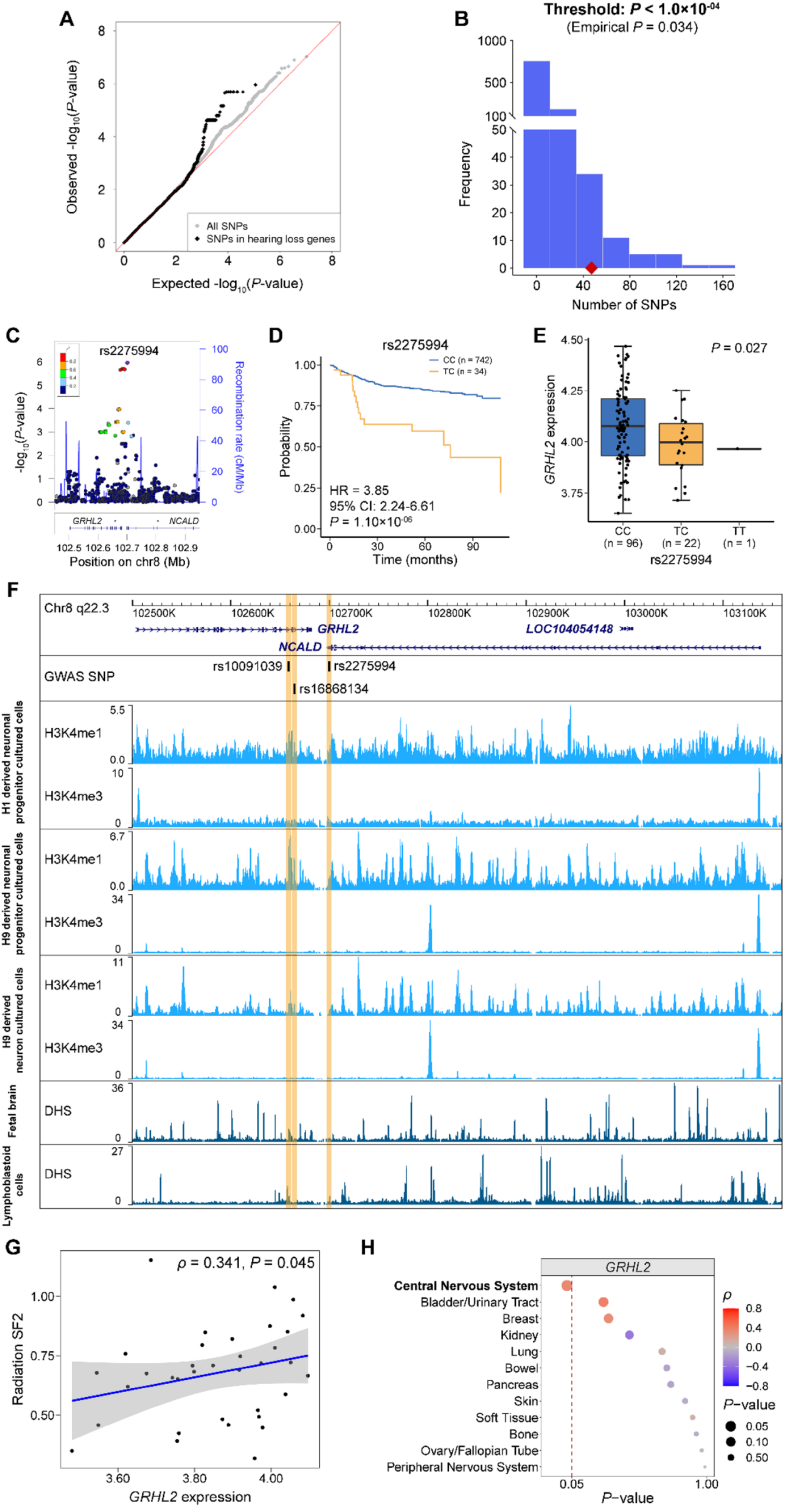
Enrichment of SNPs within 50 kb of 149 hereditary hearing loss genes among the GWAS top signals and functional annotations on *GRHL2* locus. **A** Quantile–quantile plot displays the distribution of *P*-values of overall SNPs in GWAS analysis compared with 56,040 SNPs within 50 kb of known hearing loss genes. **B** The distribution of the number of significant SNPs (*P* < 1.0 × 10^–04^) within 50 kb of hereditary deafness genes in 1000 permutation shuffles. The red diamond shows the observed number of SNPs within deafness genes exceeding the significant threshold. The empirical *P* is 0.034. **C** Regional plot of association results for *GRHL2* locus. The lead variant rs2275994 is marked by a purple diamond. The color of each circle indicates the linkage disequilibrium *r*^2^ with rs2275994 in 1000 Genome Asian populations. **D** The relationship between rs2275994 genotype and chemoradiation-induced hearing loss (no TT genotype was observed). **E** Boxplot of *GRHL2* expression in the temporal cortex (TCTX) of human brain by rs2275994 genotype. Data was obtained from BRAINEAC database. **F** ChIP-seq data show enrichments of histone modification marks and Dnase I hypersensitive sites in the sites of the lead variant rs2275994 and another two variants in moderate LD with it (rs10091039, *r*^2^ = 0.69; rs16868134, *r*^2^ = 0.66). **G** Scatter plot of radiosensitivity against normalized *GRHL2* gene expression in central nervous system cancer cell lines. **H** Correlations of radiation sensitivity with *GRHL2* expression in different cancer cell line types. The dashed red line indicates *P*-value of 0.05. *ρ* and *P*-value were calculated using Spearman’s rank correlation

To delineate a comprehensive overview of genetic etiology for hearing loss in cancer patients undergoing chemoradiation, we further evaluated whether the known 149 autosomal hereditary deafness genes were involved in CRIHL. The quantile–quantile plot indicated that variants in known deafness genes were more likely to correlate with CRIHL than expected by chance (Fig. 2A). Of 149 autosomal hearing loss genes tested, 118 genes had at least one SNP with *P*-value < 0.05 (Table S8). Interestingly, of 118 deafness genes, several genes have been reported to be associated with auditory neuropathy, a hearing dysfunction characterized by impaired transmission of signal to the auditory nerve by the presynaptic inner hair cells (Starr et al. 1996), such as *PJVK* (Delmaghani et al. 2006), *NARS2* (Simon et al. 2015), *SLC17A8* (Ruel et al. 2008), *DIAPH3* (Schoen et al. 2010) and *DIAPH1* (Wu et al. 2020).

The permutation-based enrichment analysis showed that SNPs within 50 kb of the 149 genes were significantly enriched among the GWAS top signals (*P* < 1.0 × 10^–04^) for CRIHL with an empirical *P*-value of 0.034 (Fig. 2B). The results of enrichment analysis were consistent across a range of *P*-value thresholds for GWAS from *P* < 5.0 × 10^–05^ to *P* < 5.0 × 10^–06^ and remained significant (for threshold: *P*_GWAS_ < 5.0 × 10^–05^, empirical *P* = 0.016; for threshold: *P*_GWAS_ < 1.0 × 10^–05^, empirical *P* = 0.033; for threshold: *P*_GWAS_ < 5.0 × 10^–06^, empirical *P* = 0.027; Fig. S4). But the results showed a non-significant enrichment (empirical *P* > 0.05) for the *P*-value thresholds from *P* < 5.0 × 10^–02^ to *P* < 5.0 × 10^–04^.rs2275994, located at 18 kb downstream of *GRHL2*, was the most significant SNP within the deafness loci (HR = 3.85, 95% CI 2.24–6.61, *P* = 1.10 × 10^–06^; Fig. 2C and Table S9). Patients carrying the risk allele (T) at rs2275994 were more likely to develop hearing loss than those with the non-risk allele (C) (Fig. 2D). This variant has a relatively low CADD score of 7.12 (Table S10). eQTL analysis in human brain tissues (such as the temporal cortex) showed that the risk allele at rs2275994 was correlated with lower expression of *GRHL2* (Fig. 2E and Table S11). Similar eQTL associations between *GRHL2* expression and another two variants (rs10091039 and rs16868134) in moderate LD with rs2275994 (*r*^2^ = 0.69 and 0.66, respectively) were also found in multiple brain tissues (Fig. S5 and Table S11). Additionally, rs2275994 resided in the DHS peaks in lymphoblastoid cells, and rs10091039 was located within the DHS peaks in fetal brain tissues, and rs10091039 and rs16868134 were located within the regions embracing enhancer histone marks (H3K4me1) in neuron cultured cells (Fig. 2F). These results suggested that rs2275994, rs10091039 and rs16868134 might modulate the expression of *GRHL2* by affecting chromatin accessibility, or enhancer activity to influence the risk of CRIHL.

Using cisplatin and radiation cytotoxicity analysis, we found that lower expression levels of *GRHL2* were significantly correlated with lower radiation SF2 (that is, lower tolerance to radiation) in CNS cancer cell lines (*ρ* = 0.341, *P* = 0.045; Fig. 2G), while the expression of *GRHL2* was not significantly associated with cisplatin sensitivity in the cell lines (*ρ* = 0.045, *P* = 0.768), indicative of a protective effect of *GRHL2* on radiation-related damage. Similarly, the significant association between radiosensitivity and *GRHL2* expression was specifically exited in tumor cell lines of CNS origin instead of other tissue origins (Fig. 2H).

In short, carriers with the minor allele (T) at rs2275994 would have lower *GRHL2* expression and lower radiation tolerance, being susceptible to radiation-induced damage, which may partially explain the increased hearing loss risk for patients carrying the risk allele at rs2275994 in the GWAS analysis.

### Stratified analysis

To examine the robustness of the association of ototoxicity risk with the lead variants (rs1050851 and rs2275994) identified above, we conducted stratified analysis by various clinical subgroups, such as by different groups of age (< 50 and ≥ 50 years), sex (male and female), T stage (T1–T2 and T3–T4), pre-treatment EBV DNA levels (< 4000 and ≥ 4000 copies/ml), treatment modality (CCRT alone and CCRT + IC/AC), primary tumor dose (< 70.2 and ≥ 70.2 Gy) and concurrent cisplatin dose (< 155 and ≥ 155 mg/m^2^). We found the associations between the risk of hearing loss and rs1050851 or rs2275994 were relatively stable and still remained significant within most of clinical subgroups (Fig. S6).

### Pathway analysis

**Fig. 3:**
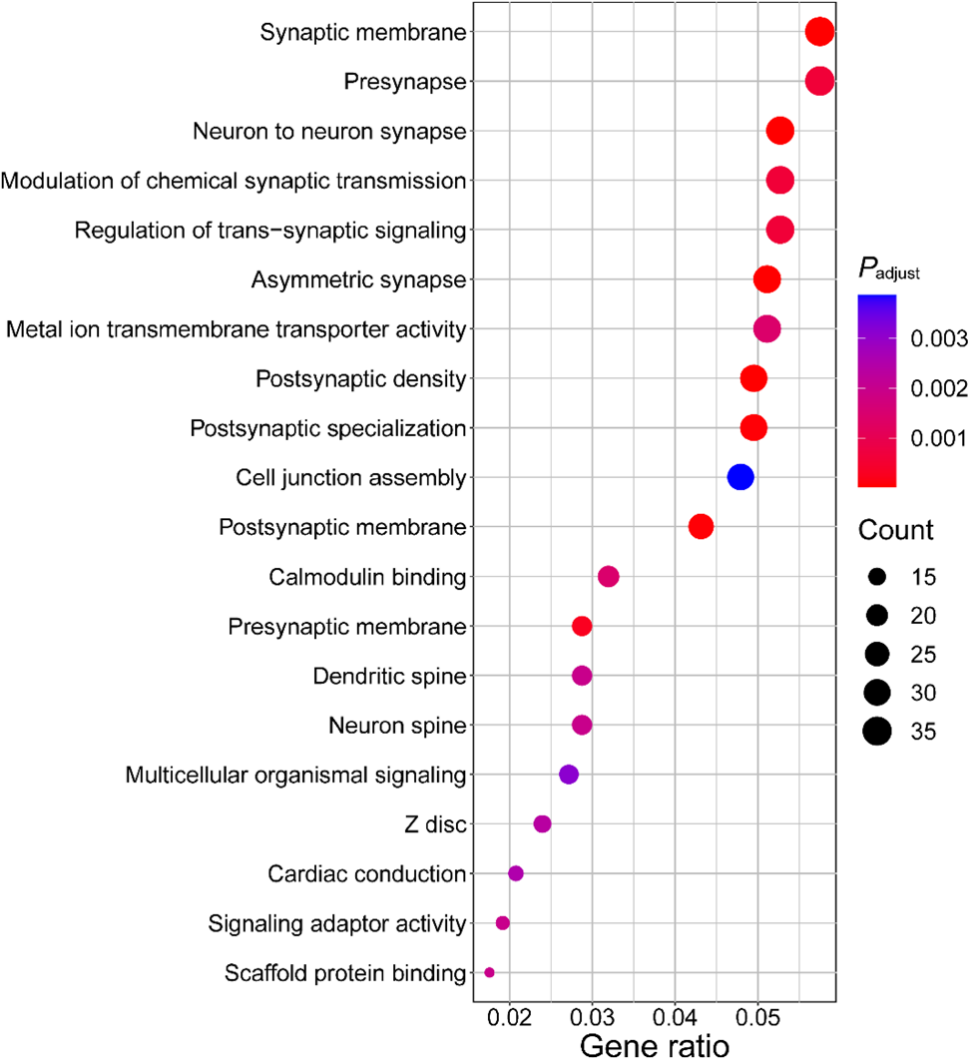
Pathway enrichment analysis based on Gene Ontology (GO) database. “Gene ratio” refers to the percentage of total candidate genes in the given pathway. All 799 candidate genes that had at least one SNP with *P* < 0.001 in the GWAS analysis within their 20 kb upstream/downstream regions were included for the pathway analysis

To further evaluate the potential biological mechanisms responsible for the development of CRIHL, we conducted pathway enrichment analysis using 799 candidate genes that had at least one SNP with *P* < 0.001 in the GWAS analysis within their 20-kb upstream/downstream regions. Based on GO enrichment analysis, candidate genes were enriched in the gene sets such as “synaptic membrane”, “neuron to neuron synapse” and “modulation of chemical synaptic transmission” (Fig. 3). This result indicated that synaptic signaling and neuronal connectivity might participate in the development of CRIHL.

### Predictive risk score model for chemoradiation-induced hearing loss

**Fig. 4:**
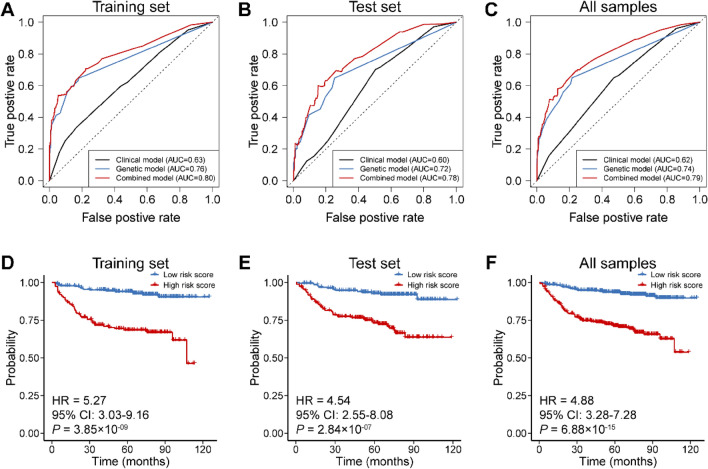
Predictive value for risk score models. **A**–**C** AUCs of different models estimating 5-years risk of chemoradiation-induced hearing loss in the training set (*n* = 389) (**A**), test set (*n* = 388) (**B**) and all samples (*n* = 777) (**C**), respectively. **D**–**F** The relationship between chemoradiation-induced hearing loss and the risk score combining genetic and clinical factors in the training set (**D**), test set (**E**) and all samples (**F**), respectively. *P* values, HRs and 95% CIs were estimated by Wald test and HRs were for high-risk score vs. low-risk score

To predict the risk of CRIHL, we developed risk score models using clinical factors only, genetic factors only, as well as integrating both clinical and genetic factors in a training set (*n* = 389), and validated their predictive values in a test set (*n* = 388). The clinical factors consisted of age at diagnosis, tumor stage, and concurrent cisplatin dose. The genetic factors included rs1050851, rs117098517, rs1485149, rs17010289, rs79938362, rs73335760, rs201061882 and rs2275994. The area under the curve (AUC) of the risk score models derived from clinical model, genetic model, and combined model in the training set were 0.63, 0.76 and 0.80, respectively (Fig. 4A). The risk score integrating genetic and clinical factors was well-replicated in the test set (AUC = 0.78; Fig. 4B), as well as in all samples (*n* = 777, AUC = 0.79; Fig. 4C).

To investigate whether the risk score integrating genetic and clinical variables can predict the development of CRIHL, we regarded the median risk score of 0.96 in the training set as the cutoff point to split all subjects into low or high-risk score groups. We found that the risk of CRIHL in patients with high-risk score was significantly higher than those with low-risk score (training set: HR = 5.27, *P* = 3.85 × 10^–09^; test set: HR = 4.54, *P* = 2.84 × 10^–07^; all samples: HR = 4.88, *P* = 6.88 × 10^–15^; Fig. 4D–F). These results suggested that the risk score combining the genetic and clinical variables could predict the development of CRIHL with a relatively good performance, which could facilitate better risk stratification.

## Discussion

Chemotherapy or radiotherapy-related hearing loss has been considered one of the most common and important survivorship concerns for cancer patients. We conducted this study to explore the clinical and genetic factors on the risk of CRIHL, and provided evidence that rs1050851 within *NFKBIA* locus was associated with CRIHL by bioinformatic functional analyses. Moreover, we found a genetic similarity between hereditary deafness and CRIHL. These findings have extended knowledge of treatment-related ototoxicity, providing opportunities to deliver more insights into the etiology of CRIHL, and new clues for preventing or alleviating the side effect of cancer treatment.

Several clinical risk factors have been reported to be associated with cisplatin or radiation-induced hearing loss (Bhandare et al. 2010; Trendowski et al. 2019), and our study further confirmed that age at diagnosis, tumor stage, and concurrent cisplatin dose were independent risk factors for CRIHL. In this study, we found that survivors who were older at diagnosis and with later T stage were more likely to experience ototoxicity after treatment, which was in accord with previous studies on NPC patients (Kwong et al. 1996; Wang et al. 2015). The survivors who were treated with a higher concurrent cisplatin dose would have a significantly higher risk of ototoxicity, while there was no significant association between induction cisplatin dose and the ototoxicity risk, demonstrating that concurrent cisplatin exerted more effects than induction or adjuvant cisplatin on hearing loss, which is also consistent with the findings from another study on hearing loss for NPC patients (Chan et al. 2009).

In addition to clinical variables, GWAS identified potential susceptible genetic variants for CRIHL. Our in-silico analysis showed that rs1050851, within the exon 2 of *NFKBIA*, was associated with cisplatin-induced hearing loss. *NFKBIA* encoding protein IκBα binds with NF-κB as an inactivated complex under resting conditions (Shih et al. 2011). When exposed to external harmful stimulation, IκBα is degraded and the activated NF-κB is released, which is involved in the inflammatory response (Vallabhapurapu and Karin 2009; Shih et al. 2011). The role of NF-κB pathway in hearing loss is complicated and multi-faceted. For example, the deficiency of NF-κB is associated with auditory nerve degeneration and increased noise-induced hearing loss in mice (Lang et al. 2006). In humans, the molecular basis of ROR1 missense mutation-caused inner ear anomalies and deafness are linked to NF-κB deficiency (Diaz-Horta et al. 2016), and this pathway also protects cochlear hair cells from aminoglycoside-induced ototoxicity (Jiang et al. 2005). However, activation of NF-κB pathway has been linked to cochlear inflammation in rats with noise-induced hearing loss (Zhang et al. 2019a). Previous studies have indicated that treatment with cisplatin in cultured auditory cells (HEI-OC1) would decrease cell viability and increase the release of proinflammatory cytokines, accompanied by the activation of the NF-κB pathway (So et al. 2007, 2008). NF-κB signaling could regulate the inflammatory processes in the central nervous system and play an important role in synaptic transmission and neuronal plasticity (O'Neill and Kaltschmidt 1997). Interestingly, our pathway enrichment analysis suggested similar pathways of synaptic signaling (such as “synaptic membrane”, “neuron to neuron synapse”, and “modulation of chemical synaptic transmission”) might participate in the development of CRIHL. Therefore, the current study provided potential clues between rs1050851 and the risk of CRIHL, and further study should be conducted to explore the underlying mechanism of *NFKBIA* in the development of chemoradiation-induced ototoxicity, such as through the neuroinflammatory processes and synaptic signaling pathway.

Moreover, we found a genetic similarity between hereditary deafness and CRIHL by permutation-based enrichment analysis. The SNPs within the regions of 149 hereditary deafness genes were more likely to be correlated with CRIHL than expected by chance and were significantly enriched among the GWAS top signals of CRIHL. rs2275994 (downstream of *GRHL2*, a known hereditary deafness gene) was the most significant SNP associated with CRIHL among the regions of 149 hereditary deafness genes. Patients carrying the risk allele (T) of rs2275994 may have lower *GRHL2* expression, which exerted risk effects on radiation damage. *GRHL2* gene encodes a transcription factor involved in epithelial differentiation, morphogenesis, and maintenance (Werth et al. 2010). Mutations in this gene, usually truncating variants, have been reported to cause autosomal dominant non-syndromic hearing loss (Peters et al. 2002; Vona et al. 2013; Trebusak Podkrajsek et al. 2021). Grhl2 protein deficiency in a zebrafish model directly reduced the expression of cldnb and epcam, junction proteins in otic epithelial cells, resulting in abnormal development of the inner ear and impaired hearing (Han et al. 2011). In addition, several lines of evidence support our assumption that hereditary deafness and CRIHL share genetic etiology. For example, variants in *WFS1*, a gene involved in both autosomal non-syndromic hearing loss and Wolfram syndrome, showed significant associations with cisplatin-induced ototoxicity in testicular cancer survivors (Wheeler et al. 2017). Mutations in hereditary deafness genes, such as *TRIOBP*, *ILDR1*, and *EYA4*, also exhibited significant correlations with age-related hearing impairment (Hoffmann et al. 2016; Wells et al. 2019).

Our study also has some limitations. First, the phenotype of hearing loss in this study was from the medical records, which are mainly based on self-reported symptoms from patients and subjective inquiry from physicians, rather than objective assessment by pure tone audiometry. A systematic review demonstrated that self-reported hearing loss based on questionnaires could accurately distinguish subjects with hearing difficulty from those without hearing difficulty (Chou et al. 2011). Previous studies utilizing self-reported hearing status have also successfully identified several genetic variants for radiation-related hearing loss in childhood cancer survivors (Trendowski et al. 2021), and age-related hearing impairment in UK Biobank populations (Wells et al. 2019). Therefore, we think hearing evaluation based on subjective sensation may be a feasible and cost-effective measurement for alternative, when audiometry test is absent. However, future work is needed to clearly elaborate or fully clarify the definition of hearing loss through pure tone audiometry, otoscopy, acoustic impedance testing, and so on, to generate more objective and reliable results. Second, the radiation dosage parameters of the inner ear or cochlea were not available in our study. It is reported that the radiation dose to the inner ear or cochlea can much better reflect the hearing loss induced by radiation therapy (Pan et al. 2005; Chan et al. 2009; Theunissen et al. 2015). However, we made some efforts to overcome this limitation. We collected the information on the prescribed total dose to the nasopharynx and included tumor stage as a covariate in GWAS analysis, which may reflect the dosage-volume parameter of the cochlea dose to some degree, since patients in later tumor stages are more likely to receive higher radiation doses to the gross tumor as well as adjacent organs including cochlea. Additionally, we did stratification analysis by tumor stage or radiation dose to the nasopharynx and found the genetic effects remained similar and robust within different subgroups (Fig. S6). Third, we think future study with larger sample size is warranted to replicate the current findings and identify more variants for CRIHL. In current study, we tested previously reported SNPs associated with cisplatin or radiation-induced hearing loss for replication. Of note, rs34533789 (within the intron of *ATXN1*), which has been reported to be associated with radiation-induced hearing loss for childhood cancer survivors (Trendowski et al. 2021), was replicated in our study (HR = 1.35, *P* value = 0.04).

In summary, our study has increased the knowledge of clinical and genetic risk factors of CRIHL, which may assist in the development of accurate prediction models to identify high-risk individuals, and contribute to precision prevention to improve quality of life for cancer survivors. However, a multicenter study with a larger sample size is needed to validate our findings and discover additional variants for CRIHL. Additional functional studies to uncover the molecular basis of CRIHL are also required, which will prompt advances in protective interventions and less ototoxic treatments.

## Supplementary Information

Below is the link to the electronic supplementary material.

Supplementary file1 (DOCX 1163 KB)

## Data Availability

The datasets used and/or analyzed during the current study are available at Research Data Deposit (RDD) public platform (http://www.researchdata.org.cn, accession number: RDDA2023873981).
