# Supplementary material for: Clinical and genome-wide association analysis of chemoradiation-induced hearing loss in nasopharyngeal carcinoma
Source: Hum Genet. 2023 Apr 16;142(6):759–72. doi: 10.1007/s00439-023-02554-0 (PMC10182145; doi:10.1007/s00439-023-02554-0)
Supplement: Supplementary file 1 — Supplementary file1 (DOCX 1163 KB) [file 439_2023_2554_MOESM1_ESM.docx]

**Supplementary Materials**

**Clinical and genome-wide association analysis of chemoradiation-induced hearing loss in nasopharyngeal carcinoma**

Yong-Qiao He, Lu-Ting Luo, Tong-Min Wang, Wen-Qiong Xue, Da-Wei Yang, Dan-Hua Li, Hua Diao, Ruo-Wen Xiao, Chang-Mi Deng, Wen-Li Zhang, Ying Liao, Yan-Xia Wu, Qiao-Ling Wang, Ting Zhou, Xi-Zhao Li, Xiao-Hui Zheng, Pei-Fen Zhang, Shao-Dan Zhang, Ye-Zhu Hu, Ying Sun, Wei-Hua Jia

Yong-Qiao He and Lu-Ting Luo contributed equally to this work.

**Corresponding author:** Wei-Hua Jia, M.D., Ph.D., Sun Yat-sen University Cancer Center, 651 Dongfeng East Road, BLDG 2, RM903, Guangzhou, Guangdong, P. R. China. 510060, Tel: 86-20-87342327, Email: [jiawh@sysucc.org.cn](mailto:jiawh@sysucc.org.cn)

**Supplementary Materials and Methods**

**Clinical data collection**

We have collected all the available variables which were established based on previous studies (Chan et al. 2009; Wang et al. 2015; Wheeler et al. 2017; El Charif et al. 2019; Wang et al. 2022), including age at diagnosis, sex, T stage, N stage, overall stage, pre-treatment EBV DNA levels, treatment modality, primary tumor dose, induction cisplatin dose, concurrent cisplatin dose, smoking, drinking, hypertension, and diabetes status. All these clinical factors were retrospectively abstracted from electronic medical records. All subjects were restaged by two experienced head and neck oncologists according to the Union for International Cancer Control/American Joint Commission on Cancer (UICC/AJCC) 2017 staging criteria.

**Functional annotations**

CADD (Combined Annotation-Dependent Depletion) scores (Kircher et al. 2014) and RegulomeDB scores (Boyle et al. 2012) were annotated to evaluate the overall deleteriousness and regulatory potential of variants (r^2^ ≥ 0.6 with the lead variants), respectively.

To explore the functional variants that reside in regions of histone marks for active promoters or enhancers, or open chromatin, we evaluated chromatin marks of variants (r^2^ ≥ 0.6 with the lead variants) by exploiting comprehensive resources. ChIP-seq data for histone modification markers (H3K27ac, H3K4me1 and H3K4me3) from 15 tissues and cell types was obtained from the Roadmap Epigenomics program (Roadmap Epigenomics et al. 2015) and the ENCODE project (Consortium 2012), including H1 derived neuronal progenitor cultured cells, H9 derived neuronal progenitor cultured cells, H9 derived neuron cultured cells, cortex derived neurospheres, ganglion eminence derived neurospheres, brain angular gyrus, brain anterior caudate, brain cingulate gyrus, brain germinal matrix, brain hippocampus middle, brain inferior temporal lobe, brain dorsolateral prefrontal cortex, brain substantia nigra, fetal brain and lymphoblastoid cells. DNase-seq data for DNase I hypersensitive site (DHS) from H1 derived neuronal progenitor cultured cells, fetal brain and lymphoblastoid cells was also obtained from the Roadmap program and the ENCODE project.

In the expression quantitative trait loci (eQTL) analysis, we used two data sources to map variants (r^2^ ≥ 0.6 with the lead variants) to genes based on eQTL links. Firstly, we analyzed associations of these SNPs with gene expression levels in the BRAINEAC database (<http://www.braineac.org/>) (Ramasamy et al. 2014), which consists of gene expression across ten human brain regions from 134 neurologically normal donors, that is, cerebellar cortex (CRBL), frontal cortex (FCTX), hippocampus (HIPP), medulla of the inferior olivary nucleus (MEDU), occipital cortex (OCTX), putamen (PUTM), substantia nigra (SNIG), temporal cortex (TCTX), thalamus (THAL) and intralobular white matter (WHMT). Additionally, we also obtained eQTL associations from other data repositories provided by FUMA, including PsychENCODE (Wang et al. 2018), xQTLServer (Ng et al. 2017), CommonMind Consortium (Fromer et al. 2016), eQTLGen (Võsa et al. 2018), Blood eQTL browser (Westra et al. 2013), BIOS QTL browser (Zhernakova et al. 2017), GTEx v8 (Consortium 2020) and eQTL Catalogue (Kerimov et al. 2021). We retained SNP-gene pairs with nominal *P*-values ≤ 0.05.

**References**

Boyle AP, Hong EL, Hariharan M, Cheng Y, Schaub MA, Kasowski M, Karczewski KJ, Park J, Hitz BC, Weng S et al (2012) Annotation of functional variation in personal genomes using RegulomeDB. Genome Res 22: 1790-1797.

Chan SH, Ng WT, Kam KL, Lee MC, Choi CW, Yau TK, Lee AW & Chow SK (2009) Sensorineural hearing loss after treatment of nasopharyngeal carcinoma: a longitudinal analysis. Int J Radiat Oncol Biol Phys 73: 1335-1342.

Consortium EP (2012) An integrated encyclopedia of DNA elements in the human genome. Nature 489: 57-74.

Consortium GT (2020) The GTEx Consortium atlas of genetic regulatory effects across human tissues. Science 369: 1318-1330.

El Charif O, Mapes B, Trendowski MR, Wheeler HE, Wing C, Dinh PC, Jr., Frisina RD, Feldman DR, Hamilton RJ, Vaughn DJ et al (2019) Clinical and Genome-wide Analysis of Cisplatin-induced Tinnitus Implicates Novel Ototoxic Mechanisms. Clin Cancer Res 25: 4104-4116.

Fromer M, Roussos P, Sieberts SK, Johnson JS, Kavanagh DH, Perumal TM, Ruderfer DM, Oh EC, Topol A, Shah HR et al (2016) Gene expression elucidates functional impact of polygenic risk for schizophrenia. Nat Neurosci 19: 1442-1453.

Kerimov N, Hayhurst JD, Peikova K, Manning JR, Walter P, Kolberg L, Samovica M, Sakthivel MP, Kuzmin I, Trevanion SJ et al (2021) A compendium of uniformly processed human gene expression and splicing quantitative trait loci. Nat Genet 53: 1290-1299.

Kircher M, Witten DM, Jain P, O'Roak BJ, Cooper GM & Shendure J (2014) A general framework for estimating the relative pathogenicity of human genetic variants. Nat Genet 46: 310-315.

Ng B, White CC, Klein HU, Sieberts SK, McCabe C, Patrick E, Xu J, Yu L, Gaiteri C, Bennett DA et al (2017) An xQTL map integrates the genetic architecture of the human brain's transcriptome and epigenome. Nat Neurosci 20: 1418-1426.

Ramasamy A, Trabzuni D, Guelfi S, Varghese V, Smith C, Walker R, De T, Consortium UKBE, North American Brain Expression C, Coin L et al (2014) Genetic variability in the regulation of gene expression in ten regions of the human brain. Nat Neurosci 17: 1418-1428.

Roadmap Epigenomics C, Kundaje A, Meuleman W, Ernst J, Bilenky M, Yen A, Heravi-Moussavi A, Kheradpour P, Zhang Z, Wang J et al (2015) Integrative analysis of 111 reference human epigenomes. Nature 518: 317-330.

Võsa U, Claringbould A, Westra H-J, Bonder MJ, Deelen P, Zeng B, Kirsten H, Saha A, Kreuzhuber R, Kasela S et al (2018) Unraveling the polygenic architecture of complex traits using blood eQTL meta-analysis. bioRxiv.

Wang D, Liu S, Warrell J, Won H, Shi X, Navarro FCP, Clarke D, Gu M, Emani P, Yang YT et al (2018) Comprehensive functional genomic resource and integrative model for the human brain. Science 362.

Wang J, Chen YY, Tai A, Chen XL, Huang SM, Yang C, Bao Y, Li NW, Deng XW, Zhao C et al (2015) Sensorineural Hearing Loss after Combined Intensity Modulated Radiation Therapy and Cisplatin-Based Chemotherapy for Nasopharyngeal Carcinoma. Transl Oncol 8: 456-462.

Wang Y, Xiao F, Zhao Y, Mao CX, Yu LL, Wang LY, Xiao Q, Liu R, Li X, McLeod HL et al (2022) A two-stage genome-wide association study to identify novel genetic loci associated with acute radiotherapy toxicity in nasopharyngeal carcinoma. Mol Cancer 21: 169.

Westra HJ, Peters MJ, Esko T, Yaghootkar H, Schurmann C, Kettunen J, Christiansen MW, Fairfax BP, Schramm K, Powell JE et al (2013) Systematic identification of trans eQTLs as putative drivers of known disease associations. Nat Genet 45: 1238-1243.

Wheeler HE, Gamazon ER, Frisina RD, Perez-Cervantes C, El Charif O, Mapes B, Fossa SD, Feldman DR, Hamilton RJ, Vaughn DJ et al (2017) Variants in WFS1 and Other Mendelian Deafness Genes Are Associated with Cisplatin-Associated Ototoxicity. Clin Cancer Res 23: 3325-3333.

Zhernakova DV, Deelen P, Vermaat M, van Iterson M, van Galen M, Arindrarto W, van 't Hof P, Mei H, van Dijk F, Westra HJ et al (2017) Identification of context-dependent expression quantitative trait loci in whole blood. Nat Genet 49: 139-145.

**Supplementary Tables**

**Table S1.** Chemoradiation-induced hearing loss risk loci with *P* < 1.0×10^-05^ in the GWAS analysis

| SNP | CHR | POS | Major allele | Minor allele | MAF (case) | MAF (control) | HR | *P*-value | Location | Gene |
| --- | --- | --- | --- | --- | --- | --- | --- | --- | --- | --- |
| rs1050851 | 14 | 35872926 | G | A | 0.043 | 0.007 | 5.46 | 9.51×10^-08^ | exonic | *NFKBIA* |
| rs117098517 | 18 | 56986855 | C | T | 0.036 | 0.005 | 6.53 | 1.29×10^-07^ | upstream | *CPLX4* |
| rs1485149 | 3 | 8013452 | A | C | 0.069 | 0.016 | 3.78 | 2.24×10^-07^ | ncRNA_intronic | *LOC101927394* |
| rs17010289 | 2 | 124346351 | A | G | 0.089 | 0.027 | 3.30 | 2.54×10^-07^ | intergenic | *LINC01826/CNTNAP5* |
| rs79938362 | 13 | 110743126 | C | T | 0.039 | 0.005 | 5.45 | 3.91×10^-07^ | intergenic | *LINC00396/COL4A1* |
| rs73335760 | 5 | 174470036 | A | G | 0.091 | 0.031 | 2.92 | 5.35×10^-07^ | intergenic | *LINC01951/DRD1* |
| rs201061882 | 21 | 33360958 | TTC | T | 0.049 | 0.011 | 4.01 | 8.15×10^-07^ | intronic | *HUNK* |
| rs2275994 | 8 | 102700230 | C | T | 0.057 | 0.015 | 3.85 | 1.10×10^-06^ | UTR3 | *NCALD* |
| rs150941604 | 5 | 78578496 | G | T | 0.038 | 0.008 | 5.07 | 1.28×10^-06^ | intronic | *JMY* |
| rs147791384 | 12 | 116980314 | G | A | 0.062 | 0.020 | 3.31 | 1.52×10^-06^ | intergenic | *LINC00173/MAP1LC3B2* |
| rs2303832 | 2 | 179472292 | T | A | 0.176 | 0.087 | 2.17 | 1.57×10^-06^ | exonic | *TTN* |
| rs72759616 | 5 | 31960647 | T | G | 0.065 | 0.018 | 3.51 | 1.79×10^-06^ | intronic | *PDZD2* |
| rs34821216 | 9 | 33919723 | A | AT | 0.142 | 0.058 | 2.33 | 1.89×10^-06^ | UTR3 | *UBE2R2* |
| rs77982841 | 3 | 141770593 | G | A | 0.059 | 0.015 | 3.42 | 2.32×10^-06^ | intronic | *TFDP2* |
| rs184839567 | 8 | 9696613 | T | G | 0.036 | 0.007 | 5.39 | 2.46×10^-06^ | intergenic | *TNKS/LINC00599* |
| rs375399598 | 10 | 101168886 | GT | G | 0.039 | 0.009 | 4.16 | 2.53×10^-06^ | intronic | *GOT1* |
| rs78144229 | 14 | 20947158 | G | A | 0.087 | 0.025 | 2.67 | 3.59×10^-06^ | downstream | *PNP* |
| rs148673917 | 6 | 8298796 | A | G | 0.049 | 0.012 | 3.88 | 4.19×10^-06^ | intergenic | *EEF1E1-BLOC1S5 /SLC35B3* |
| rs7780370 | 7 | 45540264 | C | G | 0.352 | 0.224 | 1.79 | 4.28×10^-06^ | intergenic | *RAMP3/ADCY1* |
| rs1054765 | 2 | 8811354 | A | C | 0.258 | 0.146 | 1.86 | 4.67×10^-06^ | ncRNA_exonic | *ID2-AS1* |
| rs66773189 | 2 | 186970324 | TTCTC | T | 0.306 | 0.450 | 0.52 | 4.70×10^-06^ | intergenic | *LINC01473/ZC3H15* |
| rs75002211 | 20 | 21000138 | C | T | 0.050 | 0.011 | 3.88 | 4.85×10^-06^ | intergenic | *RALGAPA2/LINC00237* |
| rs184679167 | 16 | 56524823 | G | A | 0.042 | 0.009 | 4.32 | 4.89×10^-06^ | intronic | *BBS2* |
| rs2252728 | 10 | 135275358 | G | T | 0.348 | 0.206 | 1.78 | 5.41×10^-06^ | ncRNA_intronic | *SCART1* |
| rs117170288 | 5 | 12641583 | G | A | 0.042 | 0.008 | 3.92 | 6.89×10^-06^ | ncRNA_intronic | *LINC01194* |
| rs143334999 | 10 | 23705239 | G | A | 0.063 | 0.018 | 3.36 | 7.46×10^-06^ | intergenic | *MIR1254-2/OTUD1* |
| rs143985325 | 19 | 21091519 | T | C | 0.043 | 0.010 | 4.33 | 7.94×10^-06^ | intergenic | *ZNF626/ZNF85* |
| rs12729671 | 1 | 70148282 | C | T | 0.299 | 0.184 | 1.87 | 8.70×10^-06^ | intronic | *LRRC7* |
| rs35467655 | 17 | 72240736 | T | TA | 0.125 | 0.054 | 2.20 | 8.85×10^-06^ | intronic | *TTYH2* |
| rs10885419 | 10 | 114893956 | C | G | 0.111 | 0.240 | 0.42 | 9.75×10^-06^ | intronic | *TCF7L2* |

CHR, chromosome; POS, base pair position on chromosome based on hg19; MAF, minor allele frequency; HR, hazard ratio of minor allele to major allele.

**Table S2.** Conditional analysis of SNPs in *NFKBIA* locus after adjustment for rs1050851 in 777 NPC patients

| SNP | CHR | POS | Major allele | Minor allele | MAF (case) | MAF (control) | HR | *P*-value | r^2^ | *P*-adjust | Location | Gene |
| --- | --- | --- | --- | --- | --- | --- | --- | --- | --- | --- | --- | --- |
| rs1050851 | 14 | 35872926 | G | A | 0.043 | 0.007 | 5.46 | 9.51×10^-08^ | - | - | exonic | *NFKBIA* |
| rs3138054 | 14 | 35872307 | C | T | 0.034 | 0.006 | 4.84 | 5.89×10^-06^ | 0.78 | 0.40 | intronic | *NFKBIA* |

CHR, chromosome; POS, base pair position on chromosome based on hg19; MAF, minor allele frequency; HR, hazard ratio of minor allele to major allele; r^2^, LD with rs1050851; *P*-adjust, *P* value after adjustment for rs1050851.

**Table S3.** Associations of clinical characteristics with chemoradiation-induced hearing loss in 516 nasopharyngeal carcinoma patients

| Characteristic | non-HL  (n = 424) | HL  (n = 92) | Univariate analysis | |  | Multivariate analysis | |
| --- | --- | --- | --- | --- | --- | --- | --- |
|  |  |  | HR (95% CI) | *P*-value |  | HR (95% CI)^†^ | *P*-value^†^ |
| Age at diagnosis (years) |  |  |  |  |  |  |  |
| Median (IQR) | 41.0 (35.0-48.0) | 43.5 (34.0-50.0) | 1.01 (0.98-1.03) | 0.579 |  | - | - |
| <50 | 338 (79.7%) | 65 (70.7%) | Reference |  |  | Reference |  |
| ≥50 | 86 (20.3%) | 27 (29.3%) | 1.64 (1.05-2.57) | **0.031** |  | 1.76 (1.12-2.76) | **0.015** |
| Sex |  |  |  |  |  |  |  |
| Male | 303 (71.5%) | 60 (65.2%) | Reference |  |  | Reference |  |
| Female | 121 (28.5%) | 32 (34.8%) | 1.30 (0.85-2.00) | 0.224 |  | 1.35 (0.88-2.08) | 0.173 |
| T stage |  |  |  |  |  |  |  |
| T1-T2 | 155 (36.6%) | 26 (28.3%) | Reference |  |  | Reference |  |
| T3-T4 | 269 (63.4%) | 66 (71.7%) | 1.41 (0.90-2.22) | 0.137 |  | 1.40 (0.88-2.21) | 0.155 |
| N stage |  |  |  |  |  |  |  |
| N0-N1 | 292 (68.9%) | 62 (67.4%) | Reference |  |  | Reference |  |
| N2-N3 | 132 (31.1%) | 30 (32.6%) | 1.16 (0.75-1.80) | 0.496 |  | 1.12 (0.72-1.74) | 0.612 |
| Overall stage |  |  |  |  |  |  |  |
| I-II | 108 (25.5%) | 21 (22.8%) | Reference |  |  | Reference |  |
| III-IV | 316 (74.5%) | 71 (77.2%) | 1.19 (0.73-1.93) | 0.488 |  | 0.62 (0.23-1.66) | 0.347 |
| EBV DNA loads (copies/ml) |  |  |  |  |  |  |  |
| Median (IQR) | 2435 (0-18550) | 4280 (301-26825) | 1.00 (1.00-1.00) | 0.788 |  | 1.00 (1.00-1.00) | 0.737 |
| <4000 | 228 (53.8%) | 44 (47.8%) | Reference |  |  | Reference |  |
| ≥4000 | 182 (42.9%) | 46 (50.0%) | 1.32 (0.88-2.00) | 0.184 |  | 1.26 (0.83-1.92) | 0.280 |
| NA | 14 (3.3%) | 2 (2.2%) | 0.83 (0.20-3.45) | 0.803 |  | 0.91 (0.22-3.79) | 0.900 |
| Treatment modality |  |  |  |  |  |  |  |
| CCRT alone | 241 (56.8%) | 52 (56.5%) | Reference |  |  | Reference |  |
| CCRT+IC/AC | 183 (43.2%) | 40 (43.5%) | 0.98 (0.65-1.48) | 0.920 |  | 0.71 (0.38-1.33) | 0.289 |
| Primary tumor dose (Gy) |  |  |  |  |  |  |  |
| Median (IQR) | 70.1 (69.9-70.2) | 70.1 (69.9-70.3) | 1.07 (0.83-1.38) | 0.615 |  | 0.98 (0.75-1.28) | 0.858 |
| <70.2 | 240 (56.6%) | 47 (51.1%) | Reference |  |  | Reference |  |
| ≥70.2 | 184 (43.4%) | 45 (48.9%) | 1.20 (0.80-1.81) | 0.382 |  | 1.14 (0.75-1.72) | 0.536 |
| Induction cisplatin dose (mg/m^2^) |  |  |  |  |  |  |  |
| Median (IQR); per 100 mg/m^2^ increase | 0 (0-158) | 0 (0-171) | 1.05 (0.83-1.32) | 0.692 |  | - | - |
| <162 | 334 (78.8%) | 65 (70.7%) | Reference |  |  | Reference |  |
| ≥162 | 90 (21.2%) | 27 (29.3%) | 1.43 (0.91-2.24) | 0.118 |  | 1.47 (0.93-2.32) | 0.099 |
| Concurrent cisplatin dose (mg/m^2^) |  |  |  |  |  |  |  |
| Median (IQR); per 100 mg/m^2^ increase | 198 (160-237) | 196 (162-238) | 1.06 (0.73-1.55) | 0.765 |  | - | - |
| <155 | 66 (15.6%) | 5 (5.4%) | Reference |  |  | Reference |  |
| ≥155 | 358 (84.4%) | 87 (94.6%) | 2.85 (1.15-7.01) | **0.023** |  | 3.10 (1.25-7.65) | **0.014** |
| Smoking |  |  |  |  |  |  |  |
| No | 290 (68.4%) | 70 (76.1%) | Reference |  |  | Reference |  |
| Yes | 134 (31.6%) | 22 (23.9%) | 0.73 (0.45-1.18) | 0.204 |  | 0.69 (0.42-1.11) | 0.127 |
| Drinking |  |  |  |  |  |  |  |
| No | 367 (86.6%) | 85 (92.4%) | Reference |  |  | Reference |  |
| Yes | 57 (13.4%) | 7 (7.6%) | 0.57 (0.27-1.24) | 0.158 |  | 0.59 (0.27-1.29) | 0.188 |
| Hypertension |  |  |  |  |  |  |  |
| No | 402 (94.8%) | 85 (92.4%) | Reference |  |  | Reference |  |
| Yes | 22 (5.2%) | 7 (7.6%) | 1.49 (0.69-3.22) | 0.312 |  | 1.34 (0.62-2.91) | 0.456 |
| Diabetes |  |  |  |  |  |  |  |
| No | 417 (98.3%) | 91 (98.9%) | Reference |  |  | Reference |  |
| Yes | 7 (1.7%) | 1 (1.1%) | 0.71 (0.10-5.10) | 0.734 |  | 0.86 (0.12-6.31) | 0.883 |
| Time since therapy initiation to last follow-up (months) |  |  |  |  |  |  |  |
| Median (IQR) | 68.9 (51.9-85.4) | 75.8 (57.7-89.6) | 1.00 (0.99-1.01) | 0.846 |  | 1.00 (0.99-1.01) | 0.736 |

Abbreviations: HL, hearing loss; HR, hazard ratio; CI, confidence interval; IQR, interquartile range; CCRT, concurrent chemoradiotherapy; IC, induction chemotherapy; AC, adjuvant chemotherapy; NA, not available.

^†^ As for multivariate analysis, *P*-values, HRs and 95% CIs were estimated via Cox proportional hazard model with the covariates including age at diagnosis, T stage, induction cisplatin dose and concurrent cisplatin dose. *P*-values < 0.05 are highlighted in bold.

**Table S4.** Conditional analysis of SNPs in *NFKBIA* locus after adjustment for rs1050851 in 516 NPC patients

| SNP | CHR | POS | Major allele | Minor allele | MAF (case) | MAF (control) | HR | *P*-value | r^2^ | *P*-adjust | Location | Gene |
| --- | --- | --- | --- | --- | --- | --- | --- | --- | --- | --- | --- | --- |
| rs1050851 | 14 | 35872926 | G | A | 0.063 | 0.005 | 9.16 | 3.32×10^-11^ | - | - | exonic | *NFKBIA* |
| rs3138054 | 14 | 35872307 | C | T | 0.049 | 0.004 | 7.97 | 1.24×10^-08^ | 0.78 | 0.41 | intronic | *NFKBIA* |

CHR, chromosome; POS, base pair position on chromosome based on hg19; MAF, minor allele frequency; HR, hazard ratio of minor allele to major allele; r^2^, LD with rs1050851; *P*-adjust, *P* value after adjustment for rs1050851.

**Table S5.** Functional prediction scores for SNPs within *NFKBIA* locus

| SNP | CHR | POS | Location | Gene | r^2^ | CADD | RDB |
| --- | --- | --- | --- | --- | --- | --- | --- |
| rs1050851 | 14 | 35872926 | exonic | *NFKBIA* | - | 17.10 | NA |
| rs3138054 | 14 | 35872307 | intronic | *NFKBIA* | 0.78 | 0.73 | NA |

CHR, chromosome; POS, base pair position on chromosome based on hg19; r^2^, LD with rs1050851; CADD, CADD score which predictes deleteriousness of SNPs based on 63 functional annotations; RDB, RegulomeDB score which predictes regulatory potentiality of SNPs based on eQTLs and chromatin marks; NA, not available.

**Table S6.** eQTL links of chemoradiation-induced hearing loss risk SNPs with *NFKBIA* gene expression in BRAINEAC database

| SNP | CHR | POS | Gene | Affymetrix ID | Start | Stop | eQTL *P*-value across various brain regions | | | | | | | | | | |
| --- | --- | --- | --- | --- | --- | --- | --- | --- | --- | --- | --- | --- | --- | --- | --- | --- | --- |
|  |  |  |  |  |  |  | aveALL | CRBL | FCTX | HIPP | MEDU | OCTX | PUTM | SNIG | TCTX | THAL | WHMT |
| rs1050851 | 14 | 35872926 | *NFKBIA* | 3561043 | 35871600 | 35871733 | **0.026** | 0.780 | **0.012** | 0.052 | 0.160 | 0.120 | 0.420 | **0.037** | 0.079 | **0.022** | 0.059 |
| rs1050851 | 14 | 35872926 | *NFKBIA* | 3561044 | 35871758 | 35871844 | 0.110 | 0.750 | 0.140 | **0.013** | 0.290 | 0.820 | 0.910 | 0.100 | 0.780 | **0.044** | 0.110 |
| rs1050851 | 14 | 35872926 | *NFKBIA* | 3561046 | 35871989 | 35872061 | 0.120 | 0.510 | **0.020** | **0.026** | 0.430 | 0.290 | 0.750 | 0.067 | 0.980 | 0.091 | 0.150 |
| rs1050851 | 14 | 35872926 | *NFKBIA* | 3561050 | 35872897 | 35872981 | 0.250 | **0.021** | 0.640 | 1.000 | 0.860 | **0.026** | 0.490 | 0.860 | 0.700 | 0.710 | 0.310 |
| rs1050851 | 14 | 35872926 | *NFKBIA* | 3561053 | 35873625 | 35873780 | 0.590 | **0.036** | 0.920 | **0.015** | 0.730 | 0.052 | 0.910 | 0.420 | 0.530 | **0.019** | 0.340 |
| rs3138054 | 14 | 35872307 | *NFKBIA* | 3561043 | 35871600 | 35871733 | 0.087 | 0.990 | **0.019** | 0.093 | 0.093 | 0.440 | 0.920 | **0.025** | 0.330 | **0.009** | 0.086 |
| rs3138054 | 14 | 35872307 | *NFKBIA* | 3561046 | 35871989 | 35872061 | 0.310 | 0.620 | **0.036** | **0.032** | 0.430 | **0.044** | 0.990 | 0.058 | 0.530 | 0.150 | 0.180 |
| rs3138054 | 14 | 35872307 | *NFKBIA* | 3561050 | 35872897 | 35872981 | 0.120 | **0.025** | 0.880 | 0.950 | 0.970 | **0.011** | 0.180 | 0.610 | 0.160 | 0.890 | 0.400 |
| rs3138054 | 14 | 35872307 | *NFKBIA* | 3561053 | 35873625 | 35873780 | 0.880 | **0.015** | 0.790 | **0.037** | 0.410 | 0.053 | 0.570 | 0.520 | 0.900 | 0.064 | 0.270 |

CHR, chromosome; POS, base pair position on chromosome based on hg19; Affymetrix ID, the Probe Set ID from Affymetrix; Start, start position of the Affymetrix ID; Stop, stop position of the Affymetrix ID; aveALL, the average expression across all the tissues; CRBL, cerebellar cortex; FCTX, frontal cortex; HIPP, hippocampus; MEDU, medulla (the inferior olivary nucleus); OCTX, occipital cortex; PUTM, putamen; SNIG, substantia nigra; TCTX, temporal cortex; THAL, thalamus; WHMT, intralobular white matter. eQTL results were filtered to include SNP-gene pairs with nominal *P*-values < 0.05 in any of the brain tissues. eQTL *P*-values < 0.05 are highlighted in bold.

**Table S7.** eQTL links of chemoradiation-induced hearing loss risk SNPs with *NFKBIA* gene expression in databases provided by FUMA

| SNP | CHR | POS | Gene | Database | Tissue | Tested allele | eQTL *P*-value | eQTL FDR | Risk increasing allele | Aligned direction |
| --- | --- | --- | --- | --- | --- | --- | --- | --- | --- | --- |
| rs1050851 | 14 | 35872926 | *NFKBIA* | eQTLcatalogue | CEDAR_B-cell_CD19 | G | 4.93×10^-03^ | 1.00 | A | + |
| rs1050851 | 14 | 35872926 | *NFKBIA* | eQTLGen | eQTLGen_cis_eQTLs | A | 4.37×10^-06^ | 1.25×10^-02^ | A | + |
| rs1050851 | 14 | 35872926 | *NFKBIA* | GTEx/v8 | Muscle_Skeletal | A | 3.86×10^-02^ | NA | A | - |
| rs1050851 | 14 | 35872926 | *NFKBIA* | GTEx/v8 | Adipose_Visceral_Omentum | A | 4.91×10^-02^ | NA | A | + |
| rs3138054 | 14 | 35872307 | *NFKBIA* | eQTLcatalogue | CEDAR_B-cell_CD19 | C | 2.18×10^-02^ | 1.00 | T | + |
| rs3138054 | 14 | 35872307 | *NFKBIA* | eQTLGen | eQTLGen_cis_eQTLs | T | 3.00×10^-09^ | 2.60×10^-05^ | T | + |

CHR, chromosome; POS, base pair position on chromosome based on hg19; Tested allele: tested allele of eQTLs; Risk increasing allele: risk increasing allele obtained from input GWAS summary statistics; Aligned direction: the direction of effect to gene expression after aligning risk increasing allele of GWAS and tested allele of eQTLs. "+" when the risk increasing allele increases the expression of the gene, "-" when the risk increasing allele decreases the expression of the gene. NA, not available. SNP-gene pairs with nominal *P*-values < 0.05 in any of the selected tissues were included.

**Table S8.** Hereditary deafness genes with *P* < 0.05 in the GWAS results

| Deafness gene | SNP | CHR | POS | Major allele | Minor allele | MAF (case) | MAF (control) | HR | *P*-value |
| --- | --- | --- | --- | --- | --- | --- | --- | --- | --- |
| *GRHL2* | rs2275994 | 8 | 102700230 | C | T | 0.057 | 0.015 | 3.85 | 1.10×10^-06^ |
| *ADCY1* | rs4724408 | 7 | 45570632 | T | C | 0.489 | 0.371 | 1.73 | 6.77×10^-06^ |
| *PJVK* | rs115385731 | 2 | 179347253 | C | T | 0.076 | 0.026 | 2.81 | 2.36×10^-05^ |
| *MYO3A* | rs150476026 | 10 | 26276909 | A | G | 0.051 | 0.016 | 3.06 | 1.60×10^-04^ |
| *HGF* | rs182197093 | 7 | 81348655 | C | T | 0.064 | 0.019 | 2.50 | 2.04×10^-04^ |
| *EPS8L2* | rs569407498 | 11 | 725152 | G | A | 0.030 | 0.007 | 3.35 | 3.42×10^-04^ |
| *DCDC2* | rs9356928 | 6 | 24131335 | A | G | 0.069 | 0.163 | 0.42 | 3.47×10^-04^ |
| *SLC26A5* | rs75158890 | 7 | 103060339 | T | C | 0.208 | 0.125 | 1.74 | 3.82×10^-04^ |
| *USH2A* | rs4316346 | 1 | 216100020 | C | T | 0.303 | 0.202 | 1.60 | 4.48×10^-04^ |
| *CRYM* | rs74012158 | 16 | 21311354 | T | C | 0.303 | 0.421 | 0.63 | 4.50×10^-04^ |
| *CLDN14* | rs73204270 | 21 | 37859210 | G | A | 0.098 | 0.050 | 2.03 | 6.60×10^-04^ |
| *PDE1C* | rs77451777 | 7 | 31943585 | T | G | 0.167 | 0.099 | 1.75 | 6.68×10^-04^ |
| *SLC26A4* | rs74954166 | 7 | 107269661 | C | T | 0.095 | 0.043 | 2.05 | 6.87×10^-04^ |
| *TCOF1* | rs17656747 | 5 | 149753035 | C | T | 0.057 | 0.021 | 2.34 | 7.39×10^-04^ |
| *KARS1* | rs146962138 | 16 | 75624592 | G | A | 0.038 | 0.013 | 2.91 | 1.07×10^-03^ |
| *RDX* | rs1676536 | 11 | 110162430 | T | C | 0.151 | 0.083 | 1.75 | 1.12×10^-03^ |
| *CDH23* | rs12774735 | 10 | 73242015 | C | T | 0.273 | 0.184 | 1.56 | 1.24×10^-03^ |
| *GPSM2* | rs188844055 | 1 | 109377055 | C | T | 0.062 | 0.025 | 2.41 | 1.26×10^-03^ |
| *GAB1* | rs145478287 | 4 | 144314665 | C | G | 0.030 | 0.009 | 3.17 | 1.60×10^-03^ |
| *LARS2* | rs9825692 | 3 | 45476362 | G | A | 0.352 | 0.262 | 1.50 | 1.60×10^-03^ |
| *POLR1C* | rs79222964 | 6 | 43488144 | A | G | 0.133 | 0.081 | 1.80 | 1.80×10^-03^ |
| *PCDH15* | rs145664897 | 10 | 56393005 | G | C | 0.027 | 0.006 | 3.36 | 2.17×10^-03^ |
| *TJP2* | rs371564313 | 9 | 71874886 | G | C | 0.034 | 0.012 | 2.89 | 2.51×10^-03^ |
| *KNCQ1* | rs11022821 | 11 | 2457350 | A | G | 0.027 | 0.008 | 3.17 | 2.77×10^-03^ |
| *TSPEAR* | rs34239371 | 21 | 45876065 | C | T | 0.059 | 0.026 | 2.30 | 2.80×10^-03^ |
| *MET* | rs7798983 | 7 | 116332197 | A | G | 0.470 | 0.384 | 1.43 | 3.41×10^-03^ |
| *TBC1D24* | rs117563753 | 16 | 2512963 | C | T | 0.043 | 0.013 | 2.51 | 3.85×10^-03^ |
| *POLR1D* | rs7331489 | 13 | 28279823 | G | A | 0.158 | 0.101 | 1.65 | 3.89×10^-03^ |
| *DMXL2* | rs8038101 | 15 | 51760146 | A | G | 0.415 | 0.318 | 1.43 | 4.41×10^-03^ |
| *EYA4* | rs505227 | 6 | 133592683 | C | T | 0.168 | 0.104 | 1.63 | 4.42×10^-03^ |
| *CIB2* | rs3743081 | 15 | 78463129 | C | T | 0.063 | 0.033 | 2.15 | 4.55×10^-03^ |
| *COL9A2* | rs209591 | 1 | 40816804 | G | A | 0.057 | 0.021 | 2.06 | 4.64×10^-03^ |
| *MYH14* | rs185840021 | 19 | 50675041 | C | T | 0.042 | 0.016 | 2.45 | 4.69×10^-03^ |
| *CABP2* | rs12807265 | 11 | 67335936 | C | T | 0.038 | 0.094 | 0.40 | 4.95×10^-03^ |
| *ATP2B2* | rs1553636011 | 3 | 10346995 | G | GTAA | 0.043 | 0.013 | 2.20 | 5.02×10^-03^ |
| *DSPP* | rs6829938 | 4 | 88503874 | G | C | 0.035 | 0.087 | 0.39 | 5.06×10^-03^ |
| *GSDME* | rs140466492 | 7 | 24842631 | T | C | 0.035 | 0.011 | 2.63 | 5.63×10^-03^ |
| *TMPRSS3* | rs225357 | 21 | 43786313 | C | T | 0.042 | 0.019 | 2.39 | 5.98×10^-03^ |
| *PAX3* | rs12465747 | 2 | 223152474 | G | T | 0.413 | 0.333 | 1.42 | 6.12×10^-03^ |
| *REST* | rs17081937 | 4 | 57846553 | T | C | 0.280 | 0.201 | 1.49 | 6.26×10^-03^ |
| *SIX1* | rs12880283 | 14 | 61136145 | C | T | 0.209 | 0.149 | 1.54 | 6.50×10^-03^ |
| *LOXHD1* | rs184178563 | 18 | 44191879 | G | A | 0.047 | 0.021 | 2.31 | 6.78×10^-03^ |
| *CLIC5* | rs75950397 | 6 | 45832035 | T | G | 0.028 | 0.073 | 0.36 | 6.88×10^-03^ |
| *WHRN* | rs10817612 | 9 | 117202491 | C | A | 0.069 | 0.034 | 1.93 | 6.93×10^-03^ |
| *COL2A1* | rs11168373 | 12 | 48441593 | T | C | 0.174 | 0.111 | 1.53 | 7.04×10^-03^ |
| *ESRP1* | rs56336098 | 8 | 95644061 | G | GTA | 0.449 | 0.362 | 1.41 | 7.07×10^-03^ |
| *NLRP3* | rs4925542 | 1 | 247556117 | G | A | 0.273 | 0.353 | 0.69 | 7.09×10^-03^ |
| *COL11A2* | rs111917460 | 6 | 33089162 | C | G | 0.042 | 0.019 | 2.36 | 7.13×10^-03^ |
| *MIR96* | rs4731623 | 7 | 129398442 | G | A | 0.180 | 0.118 | 1.57 | 7.19×10^-03^ |
| *GRXCR1* | rs140185086 | 4 | 42927382 | T | G | 0.036 | 0.014 | 2.55 | 7.21×10^-03^ |
| *NARS2* | rs117201481 | 11 | 78167561 | G | A | 0.047 | 0.017 | 2.27 | 7.39×10^-03^ |
| *GJB3* | rs28647679 | 1 | 35290708 | G | A | 0.027 | 0.008 | 2.46 | 7.41×10^-03^ |
| *OTOGL* | rs67188545 | 12 | 80751188 | A | AT | 0.079 | 0.043 | 1.90 | 7.59×10^-03^ |
| *LRTOMT* | rs117013948 | 11 | 71754023 | C | A | 0.028 | 0.009 | 2.81 | 7.91×10^-03^ |
| *GAS2* | rs191273027 | 11 | 22654445 | G | C | 0.030 | 0.009 | 2.65 | 8.42×10^-03^ |
| *MYO6* | rs376899413 | 6 | 76504525 | G | A | 0.023 | 0.008 | 3.06 | 8.66×10^-03^ |
| *SIX5* | rs7257321 | 19 | 46224557 | G | T | 0.024 | 0.068 | 0.34 | 8.79×10^-03^ |
| *SLC17A8* | rs10860591 | 12 | 100822511 | T | C | 0.367 | 0.282 | 1.39 | 9.57×10^-03^ |
| *PTPRQ* | rs7978521 | 12 | 80844370 | A | G | 0.051 | 0.022 | 2.14 | 9.84×10^-03^ |
| *CCDC50* | rs1066869 | 3 | 191165260 | A | G | 0.484 | 0.411 | 1.39 | 9.91×10^-03^ |
| *MAP1B* | rs6831 | 5 | 71515616 | A | G | 0.358 | 0.445 | 0.72 | 1.01×10^-02^ |
| *LMX1A* | rs139116081 | 1 | 165194368 | A | G | 0.030 | 0.009 | 2.56 | 1.03×10^-02^ |
| *CLRN1* | rs637899 | 3 | 150600407 | C | A | 0.500 | 0.419 | 1.40 | 1.06×10^-02^ |
| *TNC* | rs7041354 | 9 | 117924294 | A | T | 0.473 | 0.435 | 1.39 | 1.07×10^-02^ |
| *EYA1* | rs13270326 | 8 | 72158985 | G | A | 0.106 | 0.060 | 1.69 | 1.12×10^-02^ |
| *GIPC3* | rs148500211 | 19 | 3587977 | G | A | 0.020 | 0.056 | 0.31 | 1.13×10^-02^ |
| *TRIOBP* | rs62236674 | 22 | 38077935 | C | T | 0.091 | 0.053 | 1.74 | 1.15×10^-02^ |
| *IFNLR1* | rs12565101 | 1 | 24487402 | C | T | 0.462 | 0.384 | 1.37 | 1.19×10^-02^ |
| *WFS1* | rs10002652 | 4 | 6310338 | A | C | 0.129 | 0.082 | 1.61 | 1.21×10^-02^ |
| *ADGRV1* | rs78634619 | 5 | 90391447 | C | T | 0.050 | 0.023 | 2.04 | 1.29×10^-02^ |
| *ESRRB* | rs143477571 | 14 | 76905712 | A | G | 0.057 | 0.032 | 1.91 | 1.38×10^-02^ |
| *USH1C* | rs34836351 | 11 | 17511301 | G | A | 0.053 | 0.030 | 2.01 | 1.39×10^-02^ |
| *SLC12A2* | rs143096524 | 5 | 127494353 | C | T | 0.024 | 0.007 | 2.84 | 1.45×10^-02^ |
| *CLDN9* | rs367588211 | 16 | 3056454 | C | T | 0.023 | 0.008 | 2.80 | 1.47×10^-02^ |
| *COL11A1* | rs77986447 | 1 | 103560886 | T | C | 0.030 | 0.011 | 2.44 | 1.53×10^-02^ |
| *HOMER2* | rs187092666 | 15 | 83501436 | G | A | 0.027 | 0.009 | 2.62 | 1.67×10^-02^ |
| *LHFPL5* | rs11348462 | 6 | 35806236 | GA | G | 0.031 | 0.010 | 2.43 | 1.67×10^-02^ |
| *SYNE4* | rs55849299 | 19 | 36546383 | C | G | 0.239 | 0.175 | 1.41 | 1.76×10^-02^ |
| *GRAP* | rs78368288 | 17 | 18927232 | A | C | 0.148 | 0.211 | 0.65 | 1.79×10^-02^ |
| *HARS2* | rs150507013 | 5 | 140024115 | TTGTG | T | 0.099 | 0.064 | 1.66 | 1.82×10^-02^ |
| *PLS1* | rs145249538 | 3 | 142448095 | G | A | 0.031 | 0.009 | 2.38 | 2.00×10^-02^ |
| *STRC* | rs138485813 | 15 | 43928794 | G | A | 0.023 | 0.009 | 2.67 | 2.02×10^-02^ |
| *METTL13* | rs1015010 | 1 | 171808551 | C | T | 0.412 | 0.489 | 0.75 | 2.08×10^-02^ |
| *MITF* | rs150514472 | 3 | 69865076 | C | T | 0.030 | 0.015 | 2.36 | 2.14×10^-02^ |
| *EDN3* | rs9679855 | 20 | 57877621 | A | G | 0.027 | 0.009 | 2.46 | 2.16×10^-02^ |
| *KCNQ4* | rs78382703 | 1 | 41248206 | T | C | 0.023 | 0.008 | 2.63 | 2.19×10^-02^ |
| *DIAPH3* | rs7342485 | 13 | 60689232 | A | G | 0.242 | 0.326 | 0.72 | 2.35×10^-02^ |
| *PNPT1* | rs4417756 | 2 | 55959964 | A | T | 0.427 | 0.498 | 0.75 | 2.40×10^-02^ |
| *SERPINB6* | rs116210820 | 6 | 2903509 | A | T | 0.057 | 0.097 | 0.54 | 2.49×10^-02^ |
| *FOXI1* | rs72828649 | 5 | 169504956 | T | A | 0.060 | 0.032 | 1.81 | 2.51×10^-02^ |
| *S1PR2* | rs118059716 | 19 | 10350977 | G | A | 0.051 | 0.025 | 1.82 | 2.63×10^-02^ |
| *COL9A1* | rs377145627 | 6 | 71061303 | TTGG | T | 0.023 | 0.011 | 2.55 | 2.84×10^-02^ |
| *CHD7* | rs75756215 | 8 | 61643023 | T | G | 0.012 | 0.041 | 0.28 | 2.92×10^-02^ |
| *COL4A3* | rs144070452 | 2 | 228204897 | G | A | 0.012 | 0.043 | 0.28 | 2.94×10^-02^ |
| *USH1G* | rs11652030 | 17 | 72876317 | C | T | 0.155 | 0.214 | 0.69 | 3.00×10^-02^ |
| *PPIP5K2* | rs149485599 | 5 | 102453956 | G | T | 0.027 | 0.015 | 2.33 | 3.13×10^-02^ |
| *HARS* | rs778582 | 5 | 140004816 | C | T | 0.133 | 0.099 | 1.49 | 3.18×10^-02^ |
| *BSND* | rs2249497 | 1 | 55456330 | T | C | 0.333 | 0.399 | 0.76 | 3.32×10^-02^ |
| *EPS8* | rs117744321 | 12 | 15863342 | T | G | 0.053 | 0.026 | 1.82 | 3.35×10^-02^ |
| *SNAI2* | rs16939033 | 8 | 49801768 | T | A | 0.008 | 0.032 | 0.22 | 3.43×10^-02^ |
| *SPNS2* | rs148077686 | 17 | 4420967 | T | C | 0.065 | 0.039 | 1.71 | 3.44×10^-02^ |
| *DIAPH1* | rs28655920 | 5 | 141034183 | A | G | 0.127 | 0.096 | 1.51 | 3.54×10^-02^ |
| *SEMA3E* | rs12672058 | 7 | 82954616 | G | C | 0.355 | 0.294 | 1.32 | 3.61×10^-02^ |
| *MYO15A* | rs75781223 | 17 | 18019192 | C | T | 0.042 | 0.022 | 1.89 | 3.61×10^-02^ |
| *KCNE1* | rs13050198 | 21 | 35819082 | T | C | 0.117 | 0.080 | 1.48 | 3.62×10^-02^ |
| *MYH9* | rs6000213 | 22 | 36632096 | G | A | 0.036 | 0.071 | 0.49 | 3.73×10^-02^ |
| *CDC14A* | rs5776515 | 1 | 101002588 | A | AT | 0.074 | 0.044 | 1.67 | 3.80×10^-02^ |
| *MYO1A* | rs188325290 | 12 | 57483563 | G | T | 0.054 | 0.026 | 1.80 | 3.97×10^-02^ |
| *SCD5* | rs17006284 | 4 | 83673517 | G | A | 0.048 | 0.028 | 1.88 | 4.03×10^-02^ |
| *COCH* | rs78721252 | 14 | 31404118 | A | G | 0.167 | 0.212 | 0.71 | 4.18×10^-02^ |
| *TMEM132E* | rs12602813 | 17 | 32981934 | C | A | 0.163 | 0.118 | 1.40 | 4.24×10^-02^ |
| *WBP2* | rs79354765 | 17 | 73897344 | G | A | 0.042 | 0.021 | 1.89 | 4.32×10^-02^ |
| *MCM2* | rs536130745 | 3 | 127282798 | TACC | T | 0.027 | 0.012 | 2.20 | 4.40×10^-02^ |
| *SLC22A4* | rs274554 | 5 | 131724950 | C | T | 0.088 | 0.124 | 0.65 | 4.50×10^-02^ |
| *OTOA* | rs62045971 | 16 | 21746642 | G | A | 0.190 | 0.143 | 1.38 | 4.56×10^-02^ |
| *CLRN2* | rs10020740 | 4 | 17515688 | T | C | 0.117 | 0.085 | 1.48 | 4.61×10^-02^ |
| *GJB2* | rs9509086 | 13 | 20767957 | T | G | 0.470 | 0.384 | 1.28 | 4.67×10^-02^ |
| *MYO7A* | rs7121392 | 11 | 76812071 | C | A | 0.177 | 0.231 | 0.72 | 4.73×10^-02^ |

CHR, chromosome; POS, base pair position on chromosome based on hg19; MAF, minor allele frequency; HR, hazard ratio of minor allele to major allele.

**Table S9.** Conditional analysis of SNPs in *GRHL2* locus after adjustment for rs2275994 in 777 NPC patients

| SNP | CHR | POS | Major allele | Minor allele | MAF (case) | MAF (control) | HR | *P*-value | r^2^ | *P*-adjust | Location | Gene |
| --- | --- | --- | --- | --- | --- | --- | --- | --- | --- | --- | --- | --- |
| rs2275994 | 8 | 102700230 | C | T | 0.057 | 0.015 | 3.85 | 1.10×10^-06^ | - | - | UTR3 | *NCALD* |
| rs10091039 | 8 | 102660252 | C | T | 0.045 | 0.015 | 2.94 | 3.87×10^-04^ | 0.69 | 0.18 | intronic | *GRHL2* |
| rs16868133 | 8 | 102663410 | A | G | 0.045 | 0.015 | 2.95 | 3.80×10^-04^ | 0.66 | 0.18 | intronic | *GRHL2* |
| rs16868134 | 8 | 102664174 | C | T | 0.045 | 0.015 | 2.95 | 3.80×10^-04^ | 0.66 | 0.18 | intronic | *GRHL2* |
| rs10093301 | 8 | 102665893 | A | C | 0.045 | 0.015 | 2.95 | 3.80×10^-04^ | 0.66 | 0.18 | intronic | *GRHL2* |
| rs10101770 | 8 | 102668312 | A | T | 0.046 | 0.014 | 3.26 | 1.12×10^-04^ | 0.73 | 0.61 | intronic | *GRHL2* |
| rs10106317 | 8 | 102669701 | C | G | 0.046 | 0.014 | 3.27 | 1.08×10^-04^ | 0.73 | 0.97 | intronic | *GRHL2* |
| rs16868159 | 8 | 102672227 | T | C | 0.046 | 0.014 | 3.26 | 1.10×10^-04^ | 0.73 | 0.97 | intronic | *GRHL2* |
| rs6996060 | 8 | 102675564 | G | A | 0.057 | 0.016 | 3.71 | 2.21×10^-06^ | 0.89 | 0.99 | intronic | *GRHL2* |
| rs10104930 | 8 | 102681820 | A | T | 0.057 | 0.016 | 3.72 | 2.04×10^-06^ | 0.92 | 1.00 | UTR3 | *GRHL2* |
| rs16868196 | 8 | 102682242 | C | T | 0.057 | 0.016 | 3.72 | 2.04×10^-06^ | 0.92 | 1.00 | downstream | *GRHL2* |
| rs141396027 | 8 | 102684067 | C | T | 0.038 | 0.012 | 2.97 | 1.01×10^-03^ | 0.60 | 0.39 | intergenic | *GRHL2/NCALD* |
| rs192243723 | 8 | 102684646 | A | C | 0.057 | 0.016 | 3.72 | 2.04×10^-06^ | 0.92 | 1.00 | intergenic | *GRHL2/NCALD* |
| rs150249158 | 8 | 102686533 | C | T | 0.057 | 0.016 | 3.72 | 2.04×10^-06^ | 0.92 | 1.00 | intergenic | *GRHL2/NCALD* |
| rs142810345 | 8 | 102686951 | C | A | 0.038 | 0.012 | 2.97 | 1.01×10^-03^ | 0.60 | 0.39 | intergenic | *GRHL2/NCALD* |
| rs375127474 | 8 | 102688459 | G | A | 0.057 | 0.016 | 3.72 | 2.04×10^-06^ | 0.92 | 1.00 | intergenic | *GRHL2/NCALD* |
| rs141273852 | 8 | 102690577 | C | T | 0.057 | 0.016 | 3.72 | 2.08×10^-06^ | 0.92 | 1.00 | intergenic | *GRHL2/NCALD* |

CHR, chromosome; POS, base pair position on chromosome based on hg19; MAF, minor allele frequency; HR, hazard ratio of minor allele to major allele; r^2^, LD with rs2275994; *P*-adjust, *P* value after adjustment for rs2275994.

**Table S10.** Functional prediction scores for SNPs within *GRHL2* locus

| SNP | CHR | POS | Location | Gene | r^2^ | CADD | RDB |
| --- | --- | --- | --- | --- | --- | --- | --- |
| rs2275994 | 8 | 102700230 | UTR3 | *NCALD* | - | 7.12 | NA |
| rs10091039 | 8 | 102660252 | intronic | *GRHL2* | 0.69 | 7.89 | 5 |
| rs16868133 | 8 | 102663410 | intronic | *GRHL2* | 0.66 | 5.46 | 6 |
| rs16868134 | 8 | 102664174 | intronic | *GRHL2* | 0.66 | 4.71 | 5 |
| rs10093301 | 8 | 102665893 | intronic | *GRHL2* | 0.66 | 0.56 | 7 |
| rs10101770 | 8 | 102668312 | intronic | *GRHL2* | 0.73 | 3.45 | 4 |
| rs10106317 | 8 | 102669701 | intronic | *GRHL2* | 0.73 | 0.81 | 6 |
| rs16868159 | 8 | 102672227 | intronic | *GRHL2* | 0.73 | 7.37 | 6 |
| rs6996060 | 8 | 102675564 | intronic | *GRHL2* | 0.89 | 0.29 | 6 |
| rs10104930 | 8 | 102681820 | UTR3 | *GRHL2* | 0.92 | 6.47 | 6 |
| rs16868196 | 8 | 102682242 | downstream | *GRHL2* | 0.92 | 1.17 | 4 |
| rs141396027 | 8 | 102684067 | intergenic | *GRHL2/NCALD* | 0.60 | 1.70 | 6 |
| rs192243723 | 8 | 102684646 | intergenic | *GRHL2/NCALD* | 0.92 | 2.45 | 7 |
| rs150249158 | 8 | 102686533 | intergenic | *GRHL2/NCALD* | 0.92 | 1.06 | 7 |
| rs142810345 | 8 | 102686951 | intergenic | *GRHL2/NCALD* | 0.60 | 2.56 | 7 |
| rs375127474 | 8 | 102688459 | intergenic | *GRHL2/NCALD* | 0.92 | 3.87 | 7 |
| rs141273852 | 8 | 102690577 | intergenic | *GRHL2/NCALD* | 0.92 | 0.49 | 6 |

CHR, chromosome; POS, base pair position on chromosome based on hg19; r^2^, LD with rs2275994; CADD, CADD score which predictes deleteriousness of SNPs based on 63 functional annotations; RDB, RegulomeDB score which predictes regulatory potentiality of SNPs based on eQTLs and chromatin marks; NA, not available.

**Table S11.** eQTL links of chemoradiation-induced hearing loss risk SNPs with *GRHL2* gene expression in BRAINEAC database

| SNP | CHR | POS | Gene | Affymetrix ID | Start | Stop | eQTL *P*-value across various brain regions | | | | | | | | | | |
| --- | --- | --- | --- | --- | --- | --- | --- | --- | --- | --- | --- | --- | --- | --- | --- | --- | --- |
|  |  |  |  |  |  |  | aveALL | CRBL | FCTX | HIPP | MEDU | OCTX | PUTM | SNIG | TCTX | THAL | WHMT |
| rs2275994 | 8 | 102700230 | *GRHL2* | t3109687 | 102504390 | 102681934 | 0.072 | 0.390 | 0.790 | 0.640 | 0.070 | 0.700 | 0.340 | **0.045** | **0.027** | 0.980 | 0.430 |
| rs2275994 | 8 | 102700230 | *GRHL2* | 3109724 | 102585948 | 102586036 | **0.034** | 0.670 | 0.870 | 0.460 | 0.320 | 0.550 | 0.240 | 0.490 | **0.008** | 0.200 | 0.860 |
| rs2275994 | 8 | 102700230 | *GRHL2* | 3109726 | 102589672 | 102589747 | 0.510 | 0.590 | 0.130 | 0.190 | 0.110 | 0.230 | **0.047** | 0.240 | 0.270 | 0.400 | 0.051 |
| rs2275994 | 8 | 102700230 | *GRHL2* | 3109748 | 102649126 | 102649156 | **0.019** | **0.040** | 0.660 | 0.450 | 0.110 | 0.780 | **0.047** | 0.170 | 0.091 | 0.610 | 0.650 |
| rs2275994 | 8 | 102700230 | *GRHL2* | 3109764 | 102676682 | 102676738 | **0.041** | 0.770 | 0.690 | **0.015** | 0.390 | 0.890 | 0.840 | 0.130 | 0.250 | 0.230 | 0.640 |
| rs2275994 | 8 | 102700230 | *GRHL2* | 3109768 | 102679038 | 102680901 | 0.630 | 0.530 | 0.870 | 0.760 | 0.430 | 0.260 | 0.930 | 0.920 | **0.018** | 0.720 | 0.750 |
| rs10091039 | 8 | 102660252 | *GRHL2* | t3109687 | 102504390 | 102681934 | 0.086 | 0.510 | 0.570 | 0.510 | 0.100 | 0.630 | 0.250 | **0.044** | **0.020** | 0.660 | 0.420 |
| rs10091039 | 8 | 102660252 | *GRHL2* | 3109724 | 102585948 | 102586036 | **0.028** | 0.590 | 0.810 | 0.360 | 0.380 | 0.450 | 0.310 | 0.550 | **0.004** | 0.170 | 0.710 |
| rs10091039 | 8 | 102660252 | *GRHL2* | 3109726 | 102589672 | 102589747 | 0.500 | 0.650 | 0.110 | 0.160 | 0.210 | 0.340 | **0.017** | 0.190 | 0.470 | 0.320 | **0.035** |
| rs10091039 | 8 | 102660252 | *GRHL2* | 3109745 | 102644465 | 102644572 | 0.390 | 0.560 | 0.120 | **0.044** | 0.310 | 0.900 | 0.170 | 0.390 | 0.160 | 0.830 | 0.320 |
| rs10091039 | 8 | 102660252 | *GRHL2* | 3109748 | 102649126 | 102649156 | **0.021** | 0.050 | 0.520 | 0.400 | 0.200 | 0.880 | **0.031** | 0.190 | **0.043** | 0.510 | 0.590 |
| rs10091039 | 8 | 102660252 | *GRHL2* | 3109764 | 102676682 | 102676738 | **0.047** | 0.920 | 0.770 | **0.010** | 0.350 | 0.750 | 0.630 | 0.120 | 0.170 | 0.350 | 0.500 |
| rs10091039 | 8 | 102660252 | *GRHL2* | 3109766 | 102678825 | 102678884 | 0.550 | 0.710 | 0.570 | 0.120 | 0.150 | 0.720 | 0.310 | 0.270 | **0.026** | 0.120 | 0.140 |
| rs16868133 | 8 | 102663410 | *GRHL2* | t3109687 | 102504390 | 102681934 | 0.072 | 0.390 | 0.790 | 0.640 | 0.070 | 0.700 | 0.340 | **0.045** | **0.027** | 0.970 | 0.430 |
| rs16868133 | 8 | 102663410 | *GRHL2* | 3109724 | 102585948 | 102586036 | **0.034** | 0.670 | 0.870 | 0.460 | 0.330 | 0.550 | 0.240 | 0.490 | **0.008** | 0.200 | 0.860 |
| rs16868133 | 8 | 102663410 | *GRHL2* | 3109726 | 102589672 | 102589747 | 0.510 | 0.590 | 0.130 | 0.190 | 0.110 | 0.230 | **0.047** | 0.240 | 0.270 | 0.400 | 0.051 |
| rs16868133 | 8 | 102663410 | *GRHL2* | 3109748 | 102649126 | 102649156 | **0.019** | **0.040** | 0.660 | 0.450 | 0.110 | 0.780 | **0.047** | 0.170 | 0.091 | 0.610 | 0.650 |
| rs16868133 | 8 | 102663410 | *GRHL2* | 3109764 | 102676682 | 102676738 | **0.041** | 0.770 | 0.690 | **0.015** | 0.390 | 0.890 | 0.840 | 0.130 | 0.250 | 0.230 | 0.640 |
| rs16868133 | 8 | 102663410 | *GRHL2* | 3109768 | 102679038 | 102680901 | 0.630 | 0.530 | 0.870 | 0.760 | 0.430 | 0.260 | 0.920 | 0.920 | **0.018** | 0.720 | 0.750 |
| rs16868134 | 8 | 102664174 | *GRHL2* | t3109687 | 102504390 | 102681934 | 0.072 | 0.390 | 0.790 | 0.640 | 0.070 | 0.700 | 0.340 | **0.045** | **0.027** | 0.980 | 0.430 |
| rs16868134 | 8 | 102664174 | *GRHL2* | 3109724 | 102585948 | 102586036 | **0.034** | 0.670 | 0.870 | 0.460 | 0.320 | 0.550 | 0.240 | 0.490 | **0.008** | 0.200 | 0.860 |
| rs16868134 | 8 | 102664174 | *GRHL2* | 3109726 | 102589672 | 102589747 | 0.510 | 0.590 | 0.130 | 0.190 | 0.110 | 0.230 | **0.047** | 0.240 | 0.270 | 0.400 | 0.051 |
| rs16868134 | 8 | 102664174 | *GRHL2* | 3109748 | 102649126 | 102649156 | **0.019** | **0.040** | 0.660 | 0.450 | 0.110 | 0.780 | **0.047** | 0.170 | 0.091 | 0.610 | 0.650 |
| rs16868134 | 8 | 102664174 | *GRHL2* | 3109764 | 102676682 | 102676738 | **0.041** | 0.770 | 0.690 | **0.015** | 0.390 | 0.890 | 0.840 | 0.130 | 0.250 | 0.230 | 0.640 |
| rs16868134 | 8 | 102664174 | *GRHL2* | 3109768 | 102679038 | 102680901 | 0.630 | 0.530 | 0.870 | 0.760 | 0.430 | 0.260 | 0.930 | 0.920 | **0.018** | 0.720 | 0.750 |
| rs10093301 | 8 | 102665893 | *GRHL2* | t3109687 | 102504390 | 102681934 | 0.072 | 0.390 | 0.790 | 0.640 | 0.070 | 0.700 | 0.340 | **0.045** | **0.027** | 0.980 | 0.430 |
| rs10093301 | 8 | 102665893 | *GRHL2* | 3109724 | 102585948 | 102586036 | **0.034** | 0.670 | 0.870 | 0.460 | 0.320 | 0.550 | 0.240 | 0.490 | **0.008** | 0.200 | 0.860 |
| rs10093301 | 8 | 102665893 | *GRHL2* | 3109726 | 102589672 | 102589747 | 0.510 | 0.590 | 0.130 | 0.190 | 0.110 | 0.230 | **0.047** | 0.240 | 0.270 | 0.400 | 0.051 |
| rs10093301 | 8 | 102665893 | *GRHL2* | 3109748 | 102649126 | 102649156 | **0.019** | **0.040** | 0.660 | 0.450 | 0.110 | 0.780 | **0.047** | 0.170 | 0.091 | 0.610 | 0.650 |
| rs10093301 | 8 | 102665893 | *GRHL2* | 3109764 | 102676682 | 102676738 | **0.041** | 0.770 | 0.690 | **0.015** | 0.390 | 0.890 | 0.840 | 0.130 | 0.250 | 0.230 | 0.640 |
| rs10093301 | 8 | 102665893 | *GRHL2* | 3109768 | 102679038 | 102680901 | 0.630 | 0.530 | 0.870 | 0.760 | 0.430 | 0.260 | 0.930 | 0.920 | **0.018** | 0.720 | 0.750 |
| rs10101770 | 8 | 102668312 | *GRHL2* | t3109687 | 102504390 | 102681934 | 0.072 | 0.390 | 0.790 | 0.640 | 0.070 | 0.700 | 0.340 | **0.045** | **0.027** | 0.980 | 0.430 |
| rs10101770 | 8 | 102668312 | *GRHL2* | 3109724 | 102585948 | 102586036 | **0.034** | 0.670 | 0.870 | 0.460 | 0.320 | 0.550 | 0.240 | 0.490 | **0.008** | 0.200 | 0.860 |
| rs10101770 | 8 | 102668312 | *GRHL2* | 3109726 | 102589672 | 102589747 | 0.510 | 0.590 | 0.130 | 0.190 | 0.110 | 0.230 | **0.047** | 0.240 | 0.270 | 0.400 | 0.051 |
| rs10101770 | 8 | 102668312 | *GRHL2* | 3109748 | 102649126 | 102649156 | **0.019** | **0.040** | 0.660 | 0.450 | 0.110 | 0.780 | **0.047** | 0.170 | 0.091 | 0.610 | 0.650 |
| rs10101770 | 8 | 102668312 | *GRHL2* | 3109764 | 102676682 | 102676738 | **0.041** | 0.770 | 0.690 | **0.015** | 0.390 | 0.890 | 0.840 | 0.130 | 0.250 | 0.230 | 0.640 |
| rs10101770 | 8 | 102668312 | *GRHL2* | 3109768 | 102679038 | 102680901 | 0.630 | 0.530 | 0.870 | 0.760 | 0.430 | 0.260 | 0.930 | 0.920 | **0.018** | 0.720 | 0.750 |
| rs10106317 | 8 | 102669701 | *GRHL2* | t3109687 | 102504390 | 102681934 | 0.072 | 0.390 | 0.790 | 0.640 | 0.070 | 0.700 | 0.340 | **0.045** | **0.027** | 0.980 | 0.430 |
| rs10106317 | 8 | 102669701 | *GRHL2* | 3109724 | 102585948 | 102586036 | **0.034** | 0.670 | 0.870 | 0.460 | 0.320 | 0.550 | 0.240 | 0.490 | **0.008** | 0.200 | 0.860 |
| rs10106317 | 8 | 102669701 | *GRHL2* | 3109726 | 102589672 | 102589747 | 0.510 | 0.590 | 0.130 | 0.190 | 0.110 | 0.230 | **0.047** | 0.240 | 0.270 | 0.400 | 0.051 |
| rs10106317 | 8 | 102669701 | *GRHL2* | 3109748 | 102649126 | 102649156 | **0.019** | **0.040** | 0.660 | 0.450 | 0.110 | 0.780 | **0.047** | 0.170 | 0.091 | 0.610 | 0.650 |
| rs10106317 | 8 | 102669701 | *GRHL2* | 3109764 | 102676682 | 102676738 | **0.041** | 0.770 | 0.690 | **0.015** | 0.390 | 0.890 | 0.840 | 0.130 | 0.250 | 0.230 | 0.640 |
| rs10106317 | 8 | 102669701 | *GRHL2* | 3109768 | 102679038 | 102680901 | 0.630 | 0.530 | 0.870 | 0.760 | 0.430 | 0.260 | 0.930 | 0.920 | **0.018** | 0.720 | 0.750 |
| rs16868159 | 8 | 102672227 | *GRHL2* | t3109687 | 102504390 | 102681934 | 0.072 | 0.390 | 0.790 | 0.640 | 0.070 | 0.700 | 0.340 | **0.045** | **0.027** | 0.980 | 0.430 |
| rs16868159 | 8 | 102672227 | *GRHL2* | 3109724 | 102585948 | 102586036 | **0.034** | 0.670 | 0.870 | 0.460 | 0.320 | 0.550 | 0.240 | 0.490 | **0.008** | 0.200 | 0.860 |
| rs16868159 | 8 | 102672227 | *GRHL2* | 3109726 | 102589672 | 102589747 | 0.510 | 0.590 | 0.130 | 0.190 | 0.110 | 0.230 | **0.047** | 0.240 | 0.270 | 0.400 | 0.051 |
| rs16868159 | 8 | 102672227 | *GRHL2* | 3109748 | 102649126 | 102649156 | **0.019** | **0.040** | 0.660 | 0.450 | 0.110 | 0.780 | **0.047** | 0.170 | 0.091 | 0.610 | 0.650 |
| rs16868159 | 8 | 102672227 | *GRHL2* | 3109764 | 102676682 | 102676738 | **0.041** | 0.770 | 0.690 | **0.015** | 0.390 | 0.890 | 0.840 | 0.130 | 0.250 | 0.230 | 0.640 |
| rs16868159 | 8 | 102672227 | *GRHL2* | 3109768 | 102679038 | 102680901 | 0.630 | 0.530 | 0.870 | 0.760 | 0.430 | 0.260 | 0.930 | 0.920 | **0.018** | 0.720 | 0.750 |
| rs6996060 | 8 | 102675564 | *GRHL2* | t3109687 | 102504390 | 102681934 | 0.072 | 0.390 | 0.790 | 0.640 | 0.070 | 0.700 | 0.340 | **0.045** | **0.027** | 0.980 | 0.430 |
| rs6996060 | 8 | 102675564 | *GRHL2* | 3109724 | 102585948 | 102586036 | **0.034** | 0.670 | 0.870 | 0.460 | 0.320 | 0.550 | 0.240 | 0.490 | **0.008** | 0.200 | 0.860 |
| rs6996060 | 8 | 102675564 | *GRHL2* | 3109726 | 102589672 | 102589747 | 0.510 | 0.590 | 0.130 | 0.190 | 0.110 | 0.230 | **0.047** | 0.240 | 0.270 | 0.400 | 0.051 |
| rs6996060 | 8 | 102675564 | *GRHL2* | 3109748 | 102649126 | 102649156 | **0.019** | **0.040** | 0.660 | 0.450 | 0.110 | 0.780 | **0.047** | 0.170 | 0.091 | 0.610 | 0.650 |
| rs6996060 | 8 | 102675564 | *GRHL2* | 3109764 | 102676682 | 102676738 | **0.041** | 0.770 | 0.690 | **0.015** | 0.390 | 0.890 | 0.840 | 0.130 | 0.250 | 0.230 | 0.640 |
| rs6996060 | 8 | 102675564 | *GRHL2* | 3109768 | 102679038 | 102680901 | 0.630 | 0.530 | 0.870 | 0.760 | 0.430 | 0.260 | 0.930 | 0.920 | **0.018** | 0.720 | 0.750 |
| rs16868196 | 8 | 102682242 | *GRHL2* | t3109687 | 102504390 | 102681934 | 0.072 | 0.390 | 0.790 | 0.640 | 0.070 | 0.690 | 0.340 | **0.045** | **0.027** | 0.970 | 0.430 |
| rs16868196 | 8 | 102682242 | *GRHL2* | 3109724 | 102585948 | 102586036 | **0.034** | 0.670 | 0.870 | 0.460 | 0.320 | 0.550 | 0.230 | 0.500 | **0.008** | 0.200 | 0.860 |
| rs16868196 | 8 | 102682242 | *GRHL2* | 3109726 | 102589672 | 102589747 | 0.510 | 0.590 | 0.130 | 0.190 | 0.110 | 0.230 | **0.046** | 0.240 | 0.270 | 0.400 | 0.051 |
| rs16868196 | 8 | 102682242 | *GRHL2* | 3109748 | 102649126 | 102649156 | **0.019** | **0.039** | 0.660 | 0.450 | 0.110 | 0.780 | **0.046** | 0.170 | 0.090 | 0.610 | 0.650 |
| rs16868196 | 8 | 102682242 | *GRHL2* | 3109764 | 102676682 | 102676738 | **0.041** | 0.770 | 0.690 | **0.015** | 0.390 | 0.890 | 0.840 | 0.130 | 0.250 | 0.230 | 0.640 |
| rs16868196 | 8 | 102682242 | *GRHL2* | 3109768 | 102679038 | 102680901 | 0.640 | 0.530 | 0.870 | 0.760 | 0.430 | 0.260 | 0.930 | 0.920 | **0.018** | 0.720 | 0.750 |
| rs192243723 | 8 | 102684646 | *GRHL2* | t3109687 | 102504390 | 102681934 | 0.072 | 0.390 | 0.780 | 0.640 | 0.070 | 0.700 | 0.340 | **0.045** | **0.027** | 0.980 | 0.420 |
| rs192243723 | 8 | 102684646 | *GRHL2* | 3109724 | 102585948 | 102586036 | **0.034** | 0.670 | 0.870 | 0.460 | 0.330 | 0.550 | 0.240 | 0.500 | **0.008** | 0.200 | 0.860 |
| rs192243723 | 8 | 102684646 | *GRHL2* | 3109726 | 102589672 | 102589747 | 0.500 | 0.590 | 0.130 | 0.190 | 0.110 | 0.230 | **0.046** | 0.240 | 0.270 | 0.410 | 0.050 |
| rs192243723 | 8 | 102684646 | *GRHL2* | 3109748 | 102649126 | 102649156 | **0.019** | **0.040** | 0.660 | 0.450 | 0.110 | 0.780 | **0.047** | 0.170 | 0.091 | 0.610 | 0.650 |
| rs192243723 | 8 | 102684646 | *GRHL2* | 3109764 | 102676682 | 102676738 | **0.041** | 0.770 | 0.690 | **0.015** | 0.390 | 0.890 | 0.840 | 0.130 | 0.260 | 0.220 | 0.640 |
| rs192243723 | 8 | 102684646 | *GRHL2* | 3109768 | 102679038 | 102680901 | 0.640 | 0.530 | 0.880 | 0.760 | 0.420 | 0.260 | 0.930 | 0.920 | **0.018** | 0.720 | 0.750 |
| rs150249158 | 8 | 102686533 | *GRHL2* | t3109687 | 102504390 | 102681934 | 0.074 | 0.390 | 0.760 | 0.640 | 0.078 | 0.700 | 0.340 | **0.045** | **0.026** | 0.990 | 0.440 |
| rs150249158 | 8 | 102686533 | *GRHL2* | 3109724 | 102585948 | 102586036 | **0.033** | 0.680 | 0.860 | 0.440 | 0.330 | 0.570 | 0.250 | 0.500 | **0.007** | 0.190 | 0.890 |
| rs150249158 | 8 | 102686533 | *GRHL2* | 3109748 | 102649126 | 102649156 | **0.018** | **0.041** | 0.660 | 0.450 | 0.120 | 0.770 | **0.046** | 0.170 | 0.085 | 0.640 | 0.640 |
| rs150249158 | 8 | 102686533 | *GRHL2* | 3109764 | 102676682 | 102676738 | **0.043** | 0.780 | 0.700 | **0.016** | 0.390 | 0.890 | 0.860 | 0.130 | 0.240 | 0.230 | 0.650 |
| rs150249158 | 8 | 102686533 | *GRHL2* | 3109768 | 102679038 | 102680901 | 0.660 | 0.530 | 0.900 | 0.760 | 0.390 | 0.270 | 0.920 | 0.920 | **0.018** | 0.740 | 0.750 |
| rs141273852 | 8 | 102690577 | *GRHL2* | t3109687 | 102504390 | 102681934 | 0.071 | 0.400 | 0.780 | 0.630 | 0.066 | 0.690 | 0.330 | **0.046** | **0.028** | 0.980 | 0.420 |
| rs141273852 | 8 | 102690577 | *GRHL2* | 3109724 | 102585948 | 102586036 | **0.031** | 0.640 | 0.870 | 0.440 | 0.330 | 0.540 | 0.230 | 0.500 | **0.007** | 0.190 | 0.890 |
| rs141273852 | 8 | 102690577 | *GRHL2* | 3109726 | 102589672 | 102589747 | 0.490 | 0.590 | 0.130 | 0.190 | 0.110 | 0.230 | **0.045** | 0.240 | 0.260 | 0.420 | **0.048** |
| rs141273852 | 8 | 102690577 | *GRHL2* | 3109748 | 102649126 | 102649156 | **0.019** | **0.042** | 0.670 | 0.450 | 0.100 | 0.790 | **0.048** | 0.170 | 0.091 | 0.590 | 0.630 |
| rs141273852 | 8 | 102690577 | *GRHL2* | 3109764 | 102676682 | 102676738 | **0.041** | 0.750 | 0.690 | **0.015** | 0.390 | 0.880 | 0.840 | 0.140 | 0.270 | 0.220 | 0.640 |
| rs141273852 | 8 | 102690577 | *GRHL2* | 3109768 | 102679038 | 102680901 | 0.650 | 0.520 | 0.900 | 0.770 | 0.440 | 0.280 | 0.920 | 0.910 | **0.018** | 0.710 | 0.750 |

CHR, chromosome; POS, base pair position on chromosome based on hg19; Affymetrix ID, the Probe Set ID from Affymetrix; Start, start position of the Affymetrix ID; Stop, stop position of the Affymetrix ID; aveALL, the average expression across all the tissues; CRBL, cerebellar cortex; FCTX, frontal cortex; HIPP, hippocampus; MEDU, medulla (the inferior olivary nucleus); OCTX, occipital cortex; PUTM, putamen; SNIG, substantia nigra; TCTX, temporal cortex; THAL, thalamus; WHMT, intralobular white matter. eQTL results were filtered to include SNP-gene pairs with nominal *P*-values < 0.05 in any of the brain tissues. eQTL *P*-values < 0.05 are highlighted in bold.

**
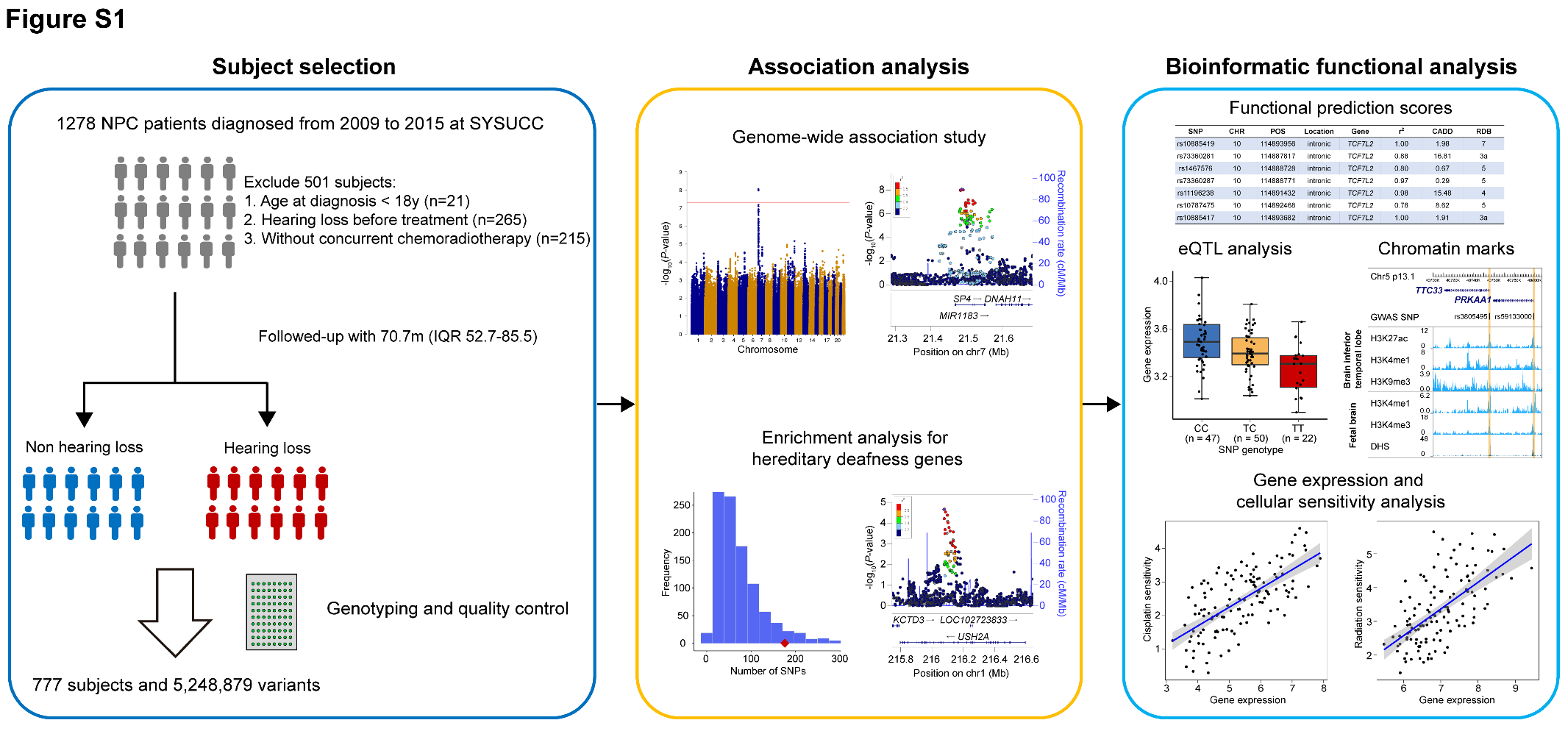
Supplementary Figures**

**Figure S1.** Study overview. NPC, nasopharyngeal carcinoma; SYSUCC, Sun Yat-sen University Cancer Center; IQR, interquartile range; eQTL, expression quantitative trait loci.


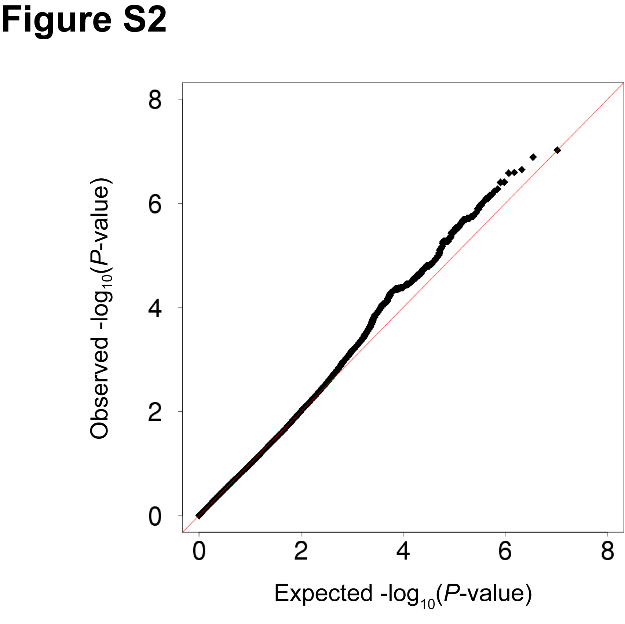


**Figure S2.** Quantile-quantile plot of GWAS results for chemoradiation-induced hearing loss. The negative logarithm (base 10) of the observed *P*-value (y axis) against the expected *P*-value (x axis) is plotted for each SNP (point).


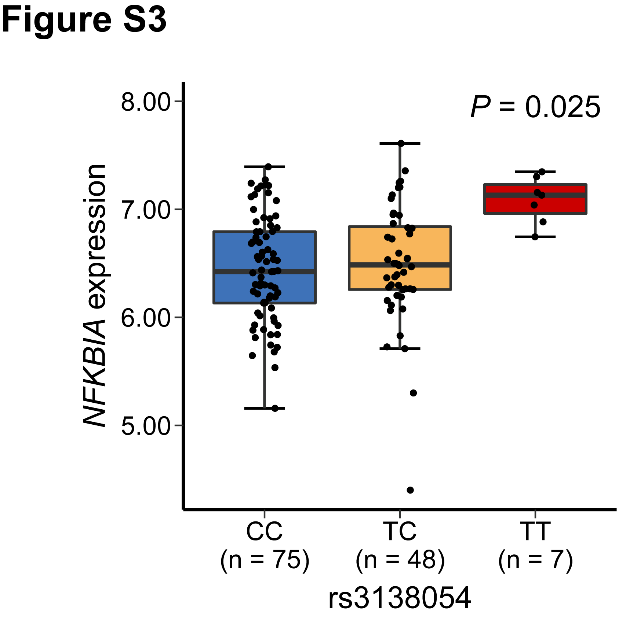


**Figure S3.** Boxplot of *NFKBIA* expression in the cerebellar cortex (CRBL) of human brain by rs3138054 genotype. Data was obtained from BRAINEAC database.


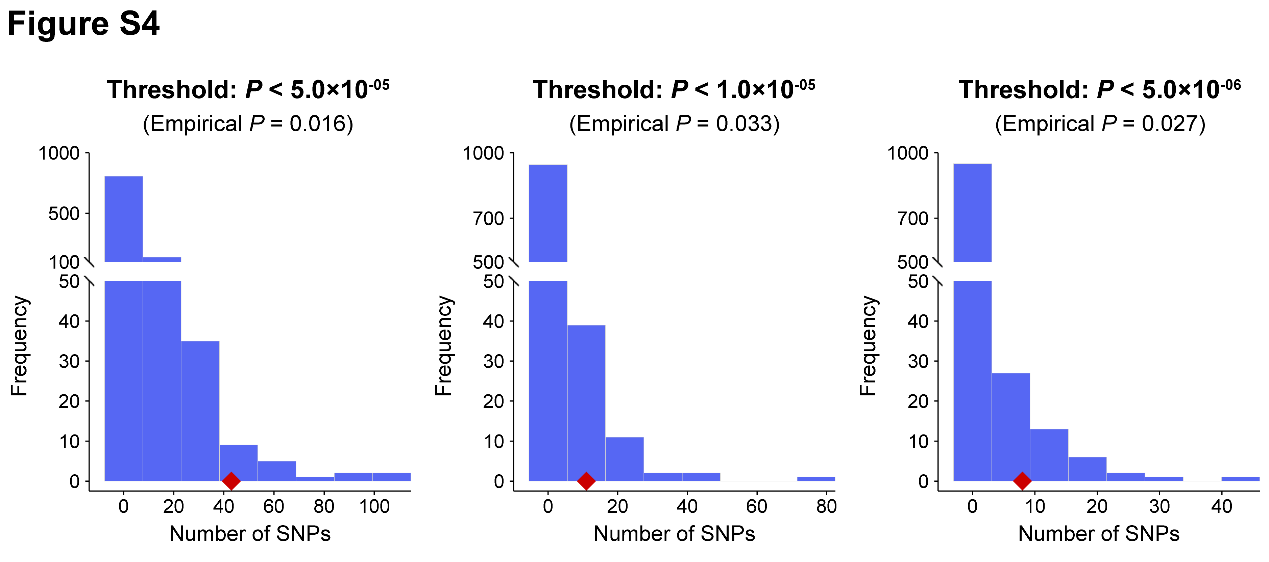


**Figure S4.** Robustness of the enrichment of SNPs within 50 kb of 149 hereditary hearing loss genes among the GWAS top signals. The distribution of the number of significant SNPs within 50 kb of known hearing loss genes at specific threshold (*P* < 5.0×10^-05^, 1.0×10^-05^, 5.0×10^-06^) in 1000 permutation shuffles. The red diamond shows the observed number of SNPs within deafness genes exceeding the significant threshold. All of the empirical *P*-values are less than 0.05 at varying thresholds.


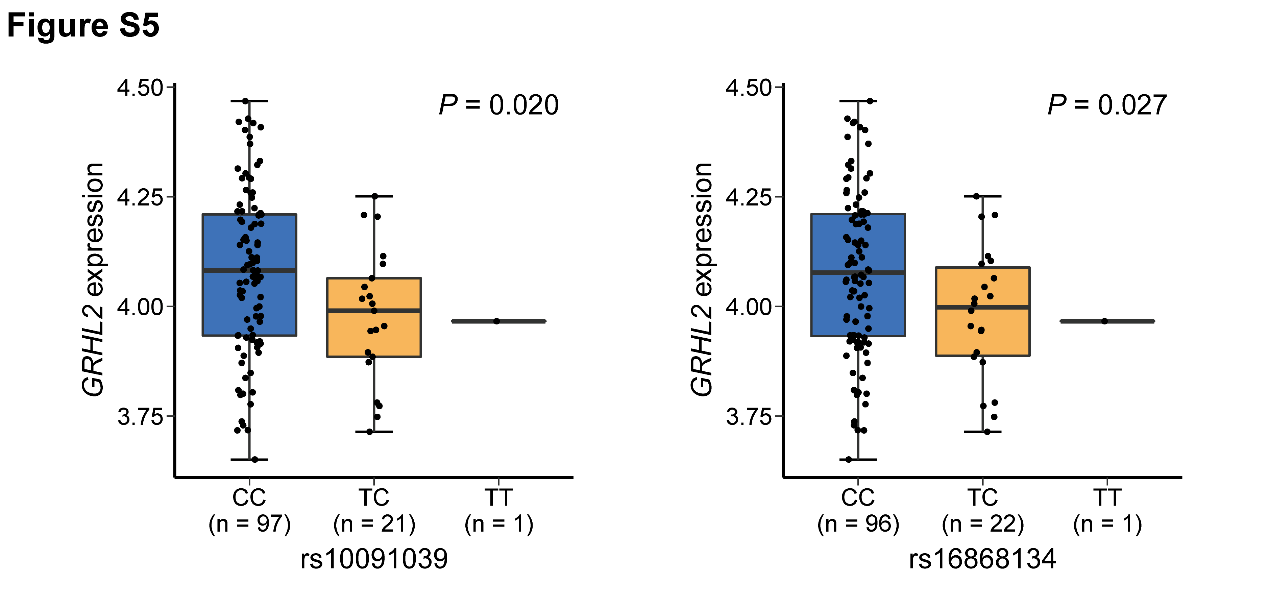


**Figure S5.** Boxplots of *GRHL2* expression in the temporal cortex (TCTX) of human brain by rs10091039 or rs16868134 genotype. Data was obtained from BRAINEAC database.


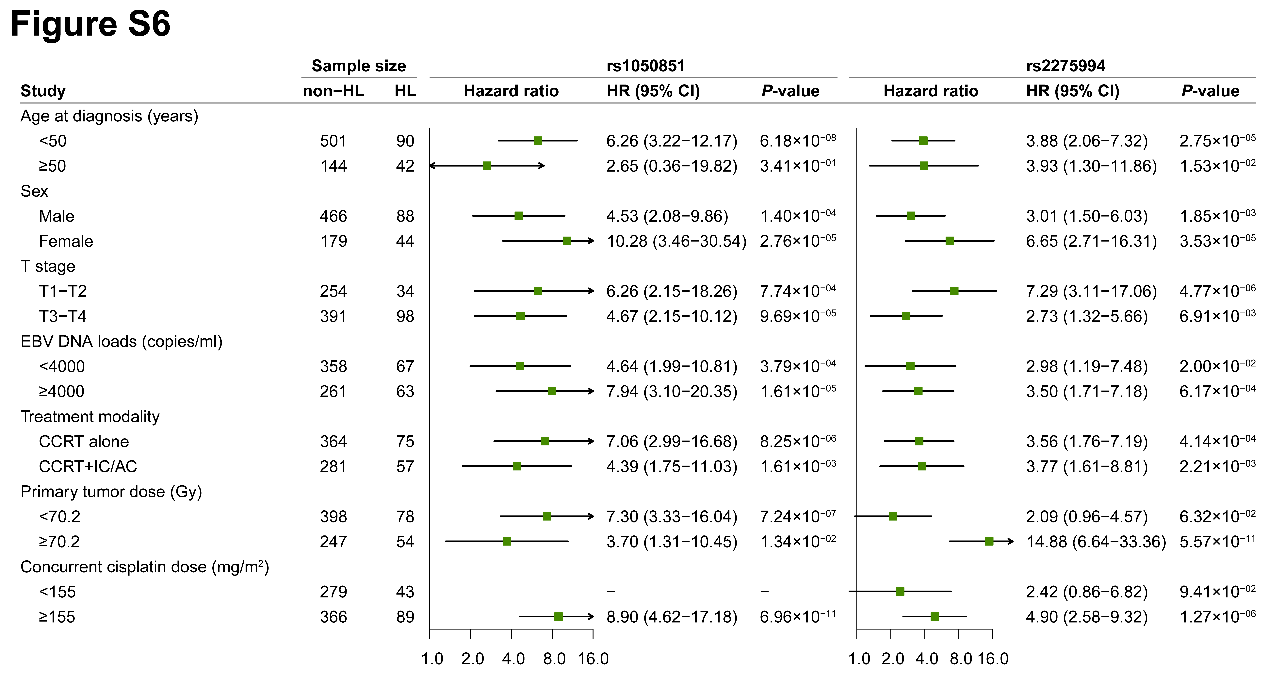


**Figure S6.** Stratified analyses of rs1050851 or rs2275994 among different subgroups. The forest plots display the results of stratified analyses of the lead variant rs1050851 or rs2275994 among different subgroups defined by age at diagnosis, sex, T stage, EBV DNA loads, treatment modality, primary tumor dose and concurrent cisplatin dose. The association could not be evaluated in the subgroup of concurrent cisplatin dose <155 mg/m^2^ for rs1050851, since hearing loss was not observed in the limited sample size of patients carrying the risk allele of rs1050851. The squares represent hazard ratios (HRs) of analyses among subgroup patients. The bars are 95% confidence intervals (CIs). HL, hearing loss; CCRT, concurrent chemoradiotherapy; IC, induction chemotherapy; AC, adjuvant chemotherapy.
